# Short-term forecasting of vegetable prices based on LSTM model—Evidence from Beijing’s vegetable data

**DOI:** 10.1371/journal.pone.0304881

**Published:** 2024-07-11

**Authors:** Qi Zhang, Weijia Yang, Anping Zhao, Xiaodong Wang, Zengfei Wang, Lin Zhang

**Affiliations:** 1 School of Mathematics and Statistics, Beijing Technology and Business University, Beijing, China; 2 Beijing Digital Agriculture Rural Promotion Center, Beijing Municipal Bureau of Agriculture and Rural Affairs, Beijing, China; Abu Dhabi University, UNITED ARAB EMIRATES

## Abstract

The vegetable sector is a vital pillar of society and an indispensable part of the national economic structure. As a significant segment of the agricultural market, accurately forecasting vegetable prices holds significant importance. Vegetable market pricing is subject to a myriad of complex influences, resulting in nonlinear patterns that conventional time series methodologies often struggle to decode. In this paper, we exploit the average daily price data of six distinct types of vegetables sourced from seven key wholesale markets in Beijing, spanning from 2009 to 2023. Upon training an LSTM model, we discovered that it exhibited exceptional performance on the test dataset. Demonstrating robust predictive performance across various vegetable categories, the LSTM model shows commendable generalization abilities. Moreover, LSTM model has a higher accuracy compared to several machine learning methods, including CNN-based time series forecasting approaches. With R^2^ score of 0.958 and MAE of 0.143, our LSTM model registers an enhancement of over 5% in forecast accuracy relative to conventional machine learning counterparts. Therefore, by predicting vegetable prices for the upcoming week, we envision this LSTM model application in real-world settings to aid growers, consumers, and policymakers in facilitating informed decision-making. The insights derived from this forecasting research could augment market transparency and optimize supply chain management. Furthermore, it contributes to the market stability and the balance of supply and demand, offering a valuable reference for the sustainable development of the vegetable industry.

## Introduction

In modern society, vegetables, as an important part of the human daily diet, have significant impacts on agricultural production, market supply, consumer purchasing power, and macroeconomic stability due to their price dynamics. The fluctuation of vegetable prices is mainly influenced by a variety of factors including supply and demand relationships, seasonal factors, climate change, and policy adjustments. With the development of information technology and big data analytics, research into vegetable price forecasting is increasingly gaining scholars’ attention. Accurate price forecasting can not only help farmers plant scientifically and allocate resources rationally but also assist governments and enterprises in developing effective market strategies, promoting the stable operation of the entire supply chain. Exploring precise methods for vegetable price forecasting has multiple benefits. It gives market participants a solid foundation for making informed decisions by providing them with comprehensive information. This helps to minimize information asymmetry and market volatility. Additionally, accurate forecasting supports the establishment of a stable supply-demand relationship. It ensures the vegetable market operates smoothly and sustains long-term growth. Moreover, it equips relevant departments with effective tools to mitigate market risks [1].

This paper introduces more complex and flexible deep learning models (LSTM model) to overcome the limitations of traditional methods. LSTM model is suitable for handling time series data, capable of capturing long-term dependencies within the data, which aids in predicting vegetable price fluctuations more accurately. As a deep learning model, LSTM can effectively capture the complex nonlinear relationships within the data, helping to enhance the reliability of the predictions. The memory cells in LSTM allow it to better retain and utilize the information from historical data, considering factors over a longer period when predicting vegetable price fluctuations. After training, LSTM model is capable of real-time predictions, assisting in timely adjustments to decision-making and strategies. Additionally, this research explores training the same model to predict the wholesale prices of multiple varieties of vegetables for the upcoming week. By using the same model, we can improve the model’s generalization, reducing the cost and complexity of building separate models for different varieties, which also enhances research efficiency and cost-effectiveness. Besides, it helps to capture the correlations and influences between different types, providing a more comprehensive market analysis and forecasting results.

The primary research contribution of this paper lies in that, through deep learning methods, an LSTM model has been constructed to predict the wholesale market prices of different types of vegetables in Beijing for the following week, filling a gap in existing research in this field. By repeatedly optimizing the LSTM model’s layers, number of hidden units, learning rate, and other hyperparameters, the best predictive performance was achieved, along with the model’s ability to generalize. The dataset used in this study differs from previous datasets as it was authoritatively collected by the government, and the selected seven wholesale markets are large enough in scale to represent the overall trend of Beijing’s wholesale market. Therefore, this study provides a new method and perspective for predicting vegetable market price fluctuations. According to the prediction results, supply chain management strategies can be adjusted, helping to optimize the production, distribution, and sales process of vegetables, improving efficiency, and reducing costs. The structure of the article is as follows: Introduction provides a clear research question and highlight the contributions of the study, introducing the research characteristics of this paper. Background and relevant literature reviews existing traditional statistical analysis forecasting methods and applications of intelligent analysis methods such as machine learning and deep learning in agricultural product market price predictions. Data selection and descriptive analysis describes the dataset used in detail, including the overall trend of vegetable prices in Beijing over the past five years and a specific analysis of the fluctuations in the prices of various types of vegetables in 2022. Model Construction introduces the basic principles of LSTM and the process of determining parameters chosen for this study. Empirical results and analysis analyzes the experimental results of LSTM on the selected dataset and its predictive performance. It also introduces other machine learning models for comparative analysis with LSTM. Conclusions and discussions summarizes the conclusions found in the paper and discusses potential future research directions.

## Background and relevant literature

Taking into account the current methods of agricultural product price forecasting both domestically and internationally, they can mainly be divided into two main categories. The first category consists of traditional statistical analysis forecasting method. These rely on mathematical statistics and econometrics theories to create models for short-term price predictions, which are based on the analysis of historical data. The second category includes intelligent analysis methods, which have gained popularity with technological advancements. Techniques such as machine learning and deep learning are now increasingly applied in predicting agricultural market prices.

### Traditional statistical analysis forecasting methods

Currently, time series methods are widely applied in the field of agricultural economics for the analysis and forecasting time series data (Jarrett F G, 1965) [2]. Traditional methods such as Moving Average (MA), Autoregressive (AR), Autoregressive Moving Average (ARMA), and Generalized Autoregressive Conditional Heteroskedasticity (GARCH) are often employed.

Fakari et al. (2013) constructed a GARCH model and found that the corn price fluctuation cycle was 21 months. They also discovered that short-term volatility in corn prices could lead to greater fluctuations in future futures prices, and that futures contracts are an effective tool for controlling corn price volatility [3]. Zhou et al. (2015) used quarterly price data from 2004 to 2014 to build an ARMA model for forecasting the prices of Chinese cabbage, cucumbers, and tomatoes. Their research indicated that vegetable price fluctuations are influenced by seasonal factors, and predicted a general upward trend in vegetable prices over the next year [4]. Xu et al. (2017) established a second-order exponential smoothing model and an ARIMA (6,1,1) model using monthly price data of carrots from Xi’an Zhuque Market between 2011 and 2015. They combined these models for short-term forecasting and suggested that agricultural product price volatility exhibits patterns different from other commodities, featuring both linear and nonlinear characteristics [5]. Razali et al. (2018) used palm oil and black pepper price data to build and compare models, identifying ARIMA (1,1,1) as the optimal model for palm oil and GARCH (1) as the most accurate for black pepper market price prediction [6]. Hong et al. (2019) pplied an ARMA-GARCH combination model to analyze the pork market index from May 2014 to May 2019. They concluded that the mismatch of supply and demand was a major factor driving the sharp increase in pork prices, and the combination model outperformed the ARMA model alone in fitting and forecasting [7]. Zhang(2019) studied the price fluctuations of mung beans and adzuki beans in China, revealing that both exhibit periodic volatility with instability, generally ranging from 2 to 4 years, with significant differences in their fluctuation characteristics [8]. Cui et al. (20121) fitted the price fluctuations of Forsythia suspense using GARCH family models. They found that the prices exhibited volatility clustering, lacking the characteristic of high risk with high returns, and that the market’s reaction to “good news” brought a greater impact on Forsythia suspense prices than the emergence of “bad news” [9]. Wang and others (2021) also used the ARIMA model to analyze wholesale garlic market price data from 2004 to 2021, forecasting the price trend for the upcoming period. The results indicated that the future market prices of garlic are likely to be relatively stable and show a downward trend [10].

### Intelligent forecasting methods such as machine learning and deep learning

Given that traditional time series models are only effective for stationary sequences, this greatly limits the widespread application and extension of time series models. With the development of machine learning technology, an increasing number of studies have begun to attempt to apply it to vegetable price forecasting, such as Back Propagation (BP) neural networks, Wavelet neural networks, Recurrent Neural Networks (RNNs), and so on.

Imagine (2011) conducted a study and forecast on the frequently fluctuating prices of meat using the Convolutional Neural Network (CNN) method and found that the CNN predictions were close to the actual price data [11]. Luo et al. (2011) used the BP neural network, genetic algorithms, and their hybrid models to make monthly predictions on the prices of shiitake mushrooms in Beijing’s Xinfadi market and provided an explanation for the theory behind various modeling methods [12]. Wang et al. (2018) used a hybrid ARIMA Support Vector Machine (SVM) model to predict the nonlinear characteristics of garlic prices, indicating that the hybrid ARIMA and SVM approach could effectively mitigate the shortcomings of single models in price prediction [13]. Wu (2019) applied the Extreme Learning Machine (ELM) model with growth limits to grain yield forecasting, significantly improving forecast accuracy [14]. Liu et al. (2019) proposed a vegetable price forecasting model combining wavelet transforms with the BP neural network, proving the effectiveness and broad applicability of machine learning models in handling vegetable price forecasting issues [15]. Hu et al. (2023) proposed research on garlic price forecasting based on deep learning, integrating a GRU network model with an attention mechanism, achieving significant success in reducing prediction errors [16].

Peng et al. (2020) integrated the BP neural network with the ARIMA model and found through comparative analysis that ARIMA was more accurate in certain cases [17]. Yu et al. (2020) proposed a short-term price forecasting combination model combining Lasso regression with the BP neural network, which showed high accuracy in cucumber price forecasting [18]. Cao et al. (2021) suggested a combined forecasting model using the X12-ARIMA model and a neural network, taking the price of lettuce in Chengdu as an example [19]. Furthermore, Liu et al. (2022) utilized multi-scale feature fusion, combining Empirical Mode Decomposition (EMD) with the Extreme Learning Machine (ELM) for the prediction of various vegetable prices, achieving good forecasting results [20].

The field-hybrid methods of metaheuristic and machine learning has become an emerging area of research. In the area of energy, Stoean (2023) introduces LSTM and Bi-LSTM models, fine-tuned with an enhanced RSA for hyperparameter tuning, to improve the accuracy of solar energy generation forecasting [21]. Gürel (2020) highlights the effectiveness of hybrid forecasting models for solar radiation, where a machine-learning algorithm, specifically a feed-forward neural network, outperforms empirical, time series, and RSM based models [22]. Similarly, Voyant (2017) emphasizes the growing trend of applying machine learning methods to solar radiation forecasting, and suggests that the incorporation of hybrid models and ensemble forecast approaches could further refine predictive performance [23]. Carrera’s comparative analysis (2020) underscores the importance of machine learning techniques in predicting photovoltaic energy generation [24]. Bacanin (2023) further illustrates the advantages of integrating metaheuristic approaches in the hyperparameter tuning of deep learning models for energy load forecasting, showing that these methods can surpass traditional tuning methods like grid search in precision [25].

In environmental study, Jovanovic (2023) explores into the explainable potential of metaheuristics-optimized XGBoost coupled with SHAP for environmental analysis. The research exemplifies how hybrid methods can uncover intricate relationships within environmental data, leading to a deeper understanding of volatile organic compounds’ behavior and aiding in the formulation of more effective environmental policies [26].

In terms of methodological research, Joaquín’s tutorial (2011) on nonparametric statistical tests provides a methodological framework for comparing the performance of evolutionary algorithms and swarm intelligence [27]. Samir (2020) develops a Genetic Algorithm-based HFS model for optimizing local and global features in handwritten word recognition [28]. Bacanin (2020) optimizes convolutional neural network hyperparameters using advanced swarm intelligence metaheuristics [29]. Bacanin (2021) proposes an advanced chaotic firefly algorithm, showcasing the capabilities of metaheuristic algorithms to enhance deep learning regularization performance [30]. Stegherr (2022) classifies metaheuristics, and contributes a structured approach to understanding and organizing the vast array of existing algorithms [31].

Additionally, Fernando (2020) analyze some of the most popular nature-inspired metaheuristics currently reported on the literature [32]. Zivkovic (2021) predicts COVID-19 cases through a hybrid machine learning model, which incorporates an adaptive neuro-fuzzy inference system with an enhanced beetle antennae search algorithm, showcasing the robustness of these hybrid methods in public health contexts [33]. Bacanin (2022) demonstrates the efficacy of hybrid methods combining metaheuristics and machine learning, such as employing Graph LSTM for pollution prediction and dragonfly algorithms for node localization in smart wireless health care systems [34]. Mihailo (2023) presents an empirical study where a LSTM network, fine-tuned by a hybrid adaptive reptile search algorithm, is used to predict Bitcoin closing prices. The approach surpasses other high-performing metaheuristic-tuned LSTM networks, illustrating the strength in financial time-series forecasting [35].

These novel research fields successfully combine machine learning and swarm intelligence approaches and proved to be able to obtain outstanding results in different areas. These studies not only confirm the effectiveness of machine learning methods in nonlinear prediction but also reveal the potential of combined models to improve forecasting accuracy.

### Review

Based on the analysis of existing literature, it can be observed that in the field of vegetable price forecasting, various models have their advantages and disadvantages. Traditional time series forecasting methods have certain limitations, such as dealing with non-stationary and nonlinear sequences. The ARMA model requires the time series to be stationary, and although differencing can help handle non-stationary time series by converting them into stationary ones, it may result in the loss of some information from the original sequence. Seasonal decomposition assumes that seasonal components are fixed and cannot adaptively accommodate different data patterns. The selection of the order for traditional models depends on experience and domain knowledge, and an order that is too high or too low can lead to inaccurate models or increased complexity. Traditional models are also relatively sensitive to noise and outliers, which can affect model accuracy if the data contains significant noise or anomalies. Fortunately, machine learning methods, especially deep learning models such as LSTM and BP neural networks, have advantages in dealing with nonlinear problems. When comparing the predictive performance of different models, researchers often use statistical metrics such as mean squared error (MSE) and mean absolute error (MAE) as evaluation criteria to quantify the accuracy of model predictions (Yuan, 2016) [36]. Therefore, this paper introduces more complex and flexible deep learning models (LSTM models) to overcome the limitations of traditional methods, and thus obtain better price prediction results.

## Data selection and descriptive analysis

### Data selection

This paper utilizes the average daily price data (in yuan per kilogram) from January 1, 2009, to September 10, 2023, from seven major wholesale markets in Beijing (Xinfadi, Yuegezhuang, Dayanglu, Shimen, Baliqiao, Shuitun, and Jinxiu Dadi wholesale markets). The dataset has the following two advantages: 1) The selected seven wholesale markets are all relatively large in scale and can reflect the overall market trend of Beijing’s wholesale markets, making them representative. 2) The data is collected over a long-time span and authoritatively collected by the government, making it suitable for the study of vegetable price fluctuations and for conducting both long-term and short-term forecasts.

In model training, from four major categories—leaf vegetables, solanaceous vegetables, storage-resistant vegetables, and edible fungi—one to two kinds of vegetables were selected from each category, thus choosing the daily price data of next six vegetables: celery, spiny cucumber, eggplant, carrot, potato, and oyster mushroom.

### Overall situation of vegetable prices

**Fig 1:**
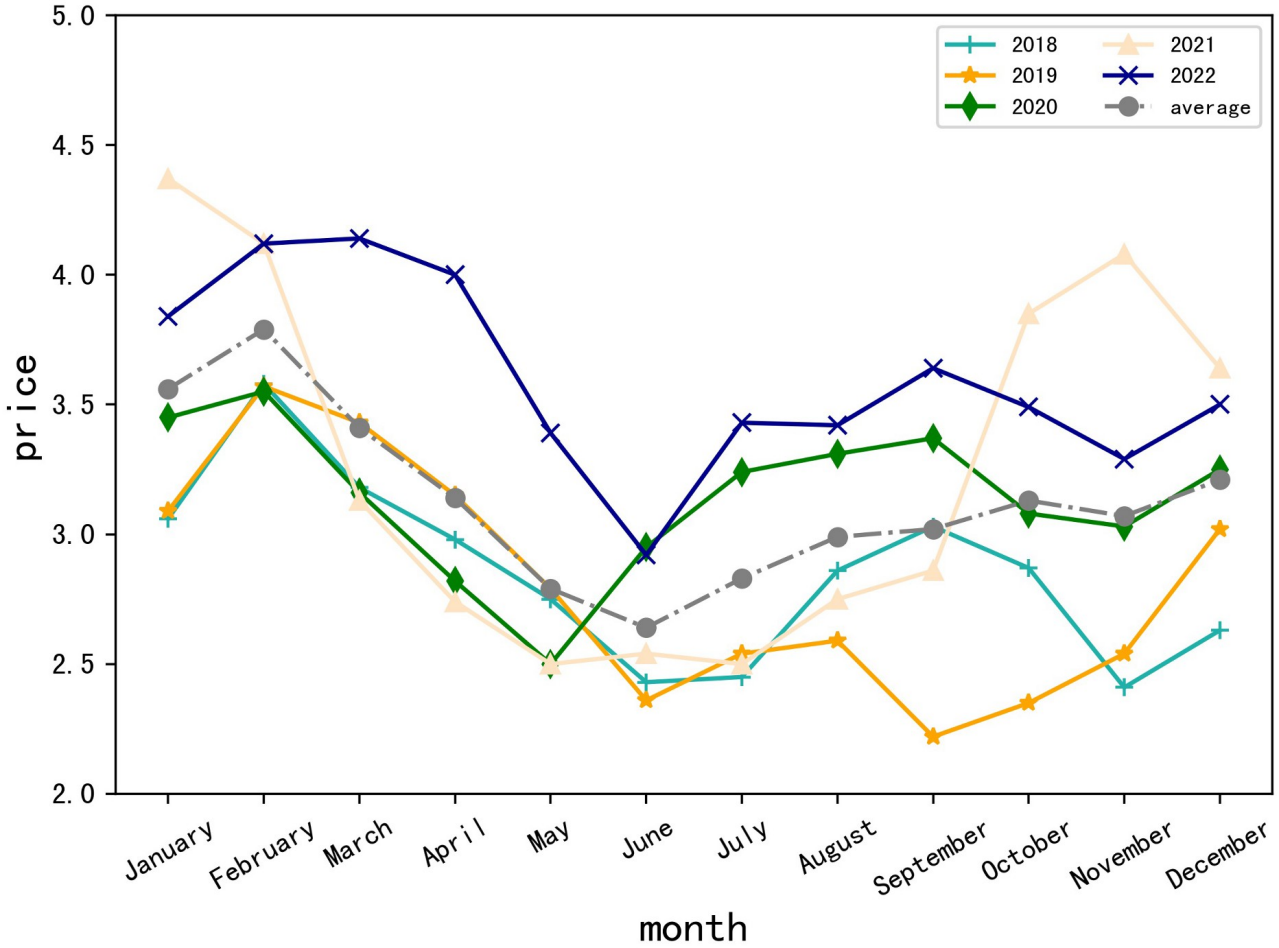
Trends in monthly vegetable prices in Beijing over the past five years.

As shown in Fig 1, the line chart presents the monthly prices of vegetables in Beijing over the past five years (2018, 2019, 2020, 2021, 2022) and the trend of the average price over these five years. The chart reveals that the overall prices of vegetables in Beijing tend to rise year by year. Moreover, there is a seasonal pattern where prices are generally higher in winter and spring, and lower in summer and fall. By comparing the data for one year after the COVID-19 pandemic (2022) with that of the previous year (2019), it’s clear that despite the year-over-year increase in vegetable prices, the monthly price trends remain largely consistent. Meanwhile, the trend in 2020 and 2022 also shows considerable consistency, and the 2022 data roughly aligns with the average price trend of the past five years. Typically, June records the lowest prices of the year, while January, February, and December experience relatively higher prices. From March to June, prices generally decline and then gradually increase thereafter. Therefore, to a certain extent, the data from 2022 can represent the fluctuation patterns of recent years.

### Price trends of six representative vegetables in 2022

To reflect the latest trends in vegetable prices, this paper selects the complete cycle data of the most recent year (i.e., 2022) and conducts a volatility analysis discussion by selecting 1–2 vegetables from each of the four major categories: leafy vegetables, solanaceous vegetables, storage-resistant vegetables, and edible fungi. Celery represents leafy vegetables; spiny cucumber and eggplant are chosen from solanaceous family; carrot and potato are the representatives of storage-resistant vegetables; and oyster mushroom is included as an example of the edible fungi category.

**Fig 2:**
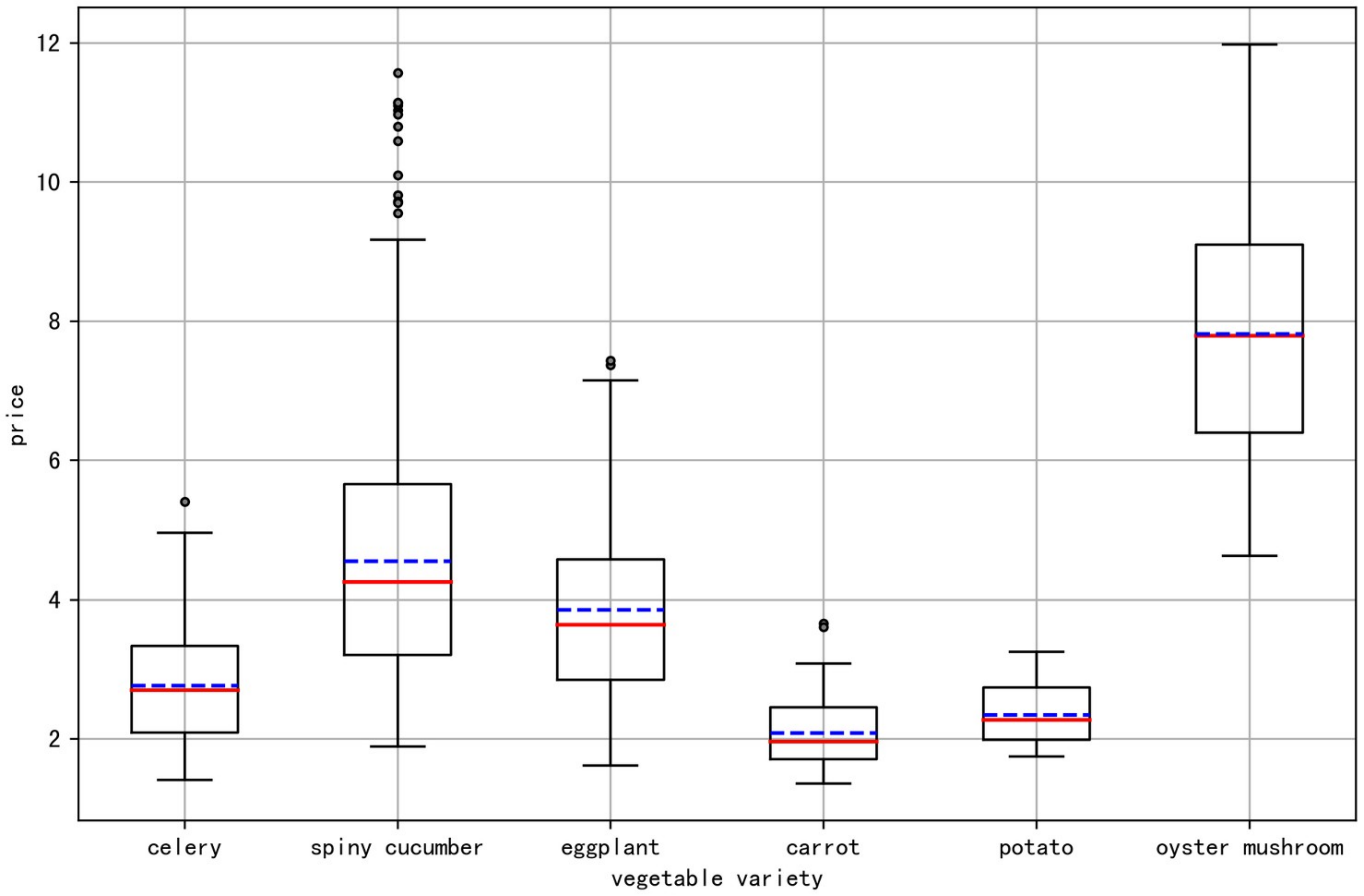
Box plot demonstration of daily prices for six types of vegetables in 2022.

Fig 2 showcases a box plot analysis of the daily prices for six vegetable in 2022, visualizing the price variations for each category. The box plots indicate minimal outliers in the price data for vegetables such as celery, eggplant, carrot, potato, and oyster mushroom, with some categories showing no outliers. In contrast, spiney cucumber prices exhibit outliers primarily around the Chinese New Year, a period known for market price volatility. These outliers are considered typical for the festive season and do not require removal from the dataset. Consequently, the dataset is deemed reliable and appropriate for the intended model training and analytical purposes.

**Fig 3:**
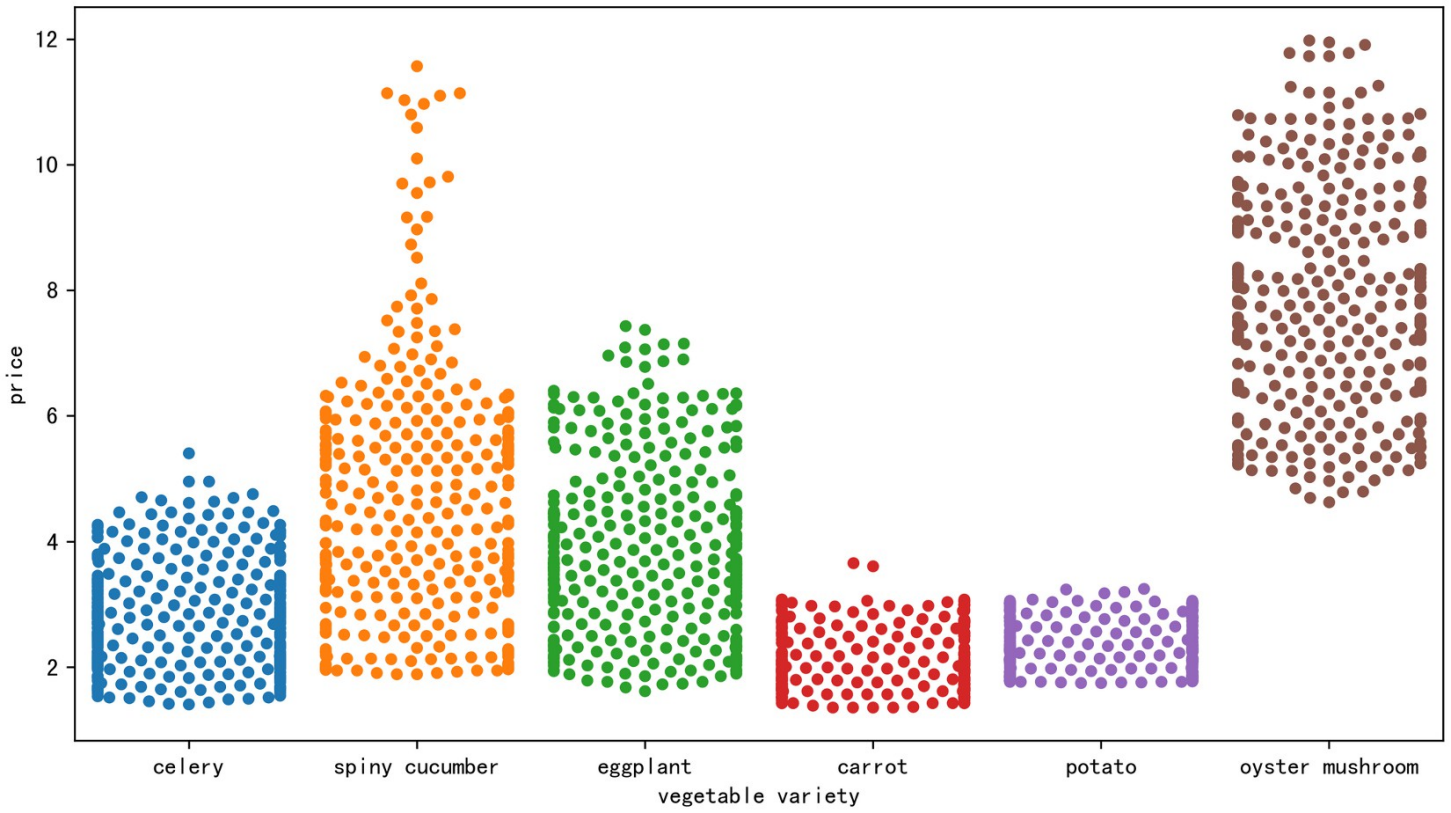
Swarm plot demonstration of daily prices for six types of vegetables in 2022.

Fig 3 also selects the daily price data from 2022 to present a swarm plot for six vegetable varieties. It can be observed that each type of vegetable has a relatively dense price range. Carrots have a relatively low price among the six vegetables, mainly concentrated between 1.5 to 3.5 yuan; mushrooms have relatively higher and more dispersed prices, ranging from 5 to 11 yuan. Carrots and potatoes, as representatives of storage-resistant vegetables, have a relatively smaller range of price fluctuations, mainly concentrated below 3.5 yuan.

The detailed analysis of six vegetables are as follows. Based on the daily price data of different varieties in 2022, the monthly average prices are calculated, and the standard deviations for each month are computed. Error bars are used to display the standard deviation results, providing a visual representation of price fluctuations.

#### There were three price troughs in the 2022 celery prices

**Fig 4:**
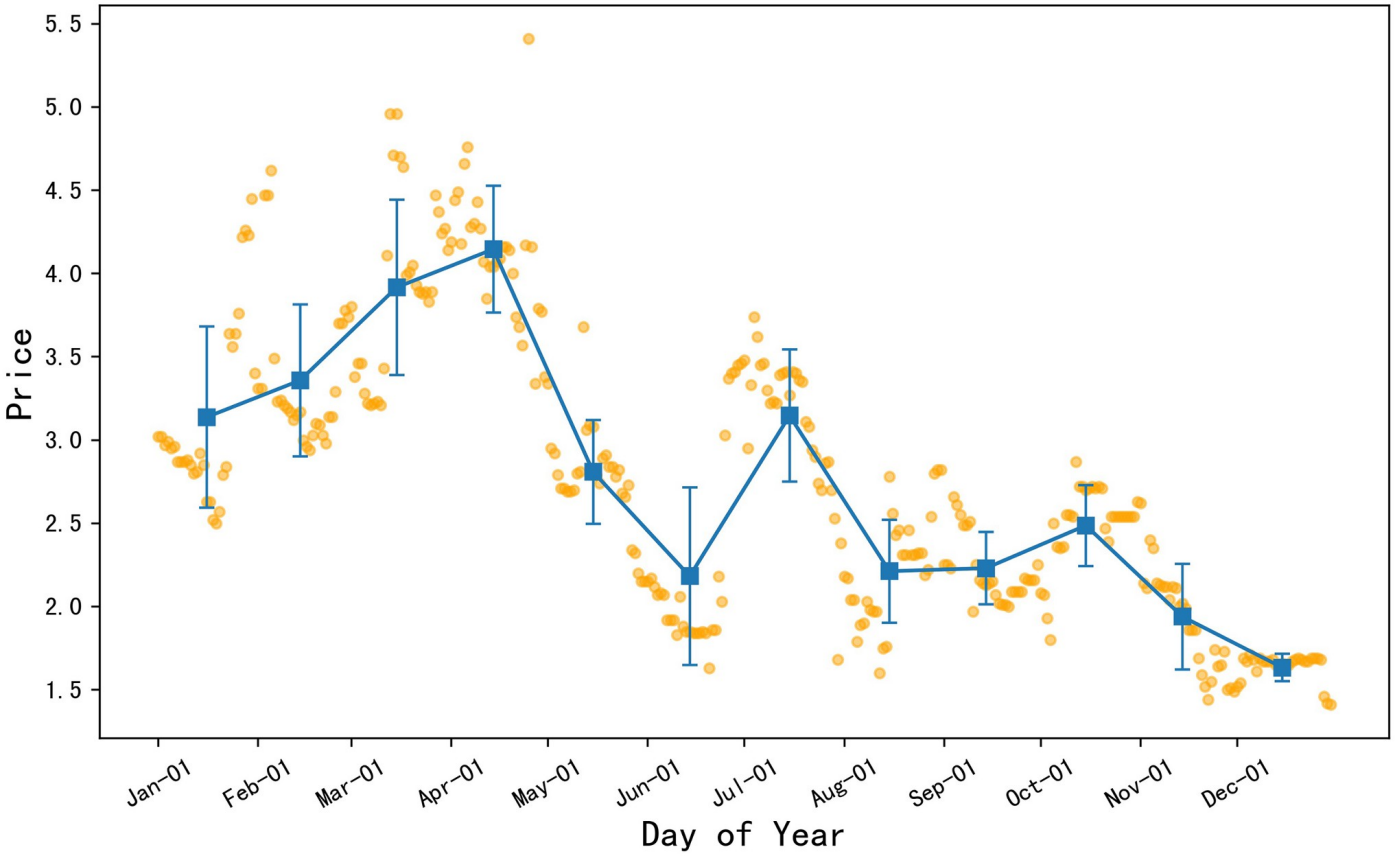
2022 daily average price chart for celery.

As illustrated in Fig 4, the orange dots represent the daily price data of celery in 2022, and the blue square points represent the monthly average price of celery. The error bars are set to the standard deviation of the monthly price data. From the chart, it can be inferred that the average prices of celery in January, February, March, and April of 2022 were relatively high, with the lowest average price occurring in December. Prices began to decline overall from mid to late April, and despite a slight rise in early to mid-July, then remained below the average levels of January, February, March, and April. According to the error bar results, celery experienced larger fluctuations within the months of January, February, and March, while the fluctuations were smaller within September, October, and December, indicating a more stable celery price during these months.

#### The overall trend of 2022 spiny cucumber prices showed a “W-shaped” pattern

**Fig 5:**
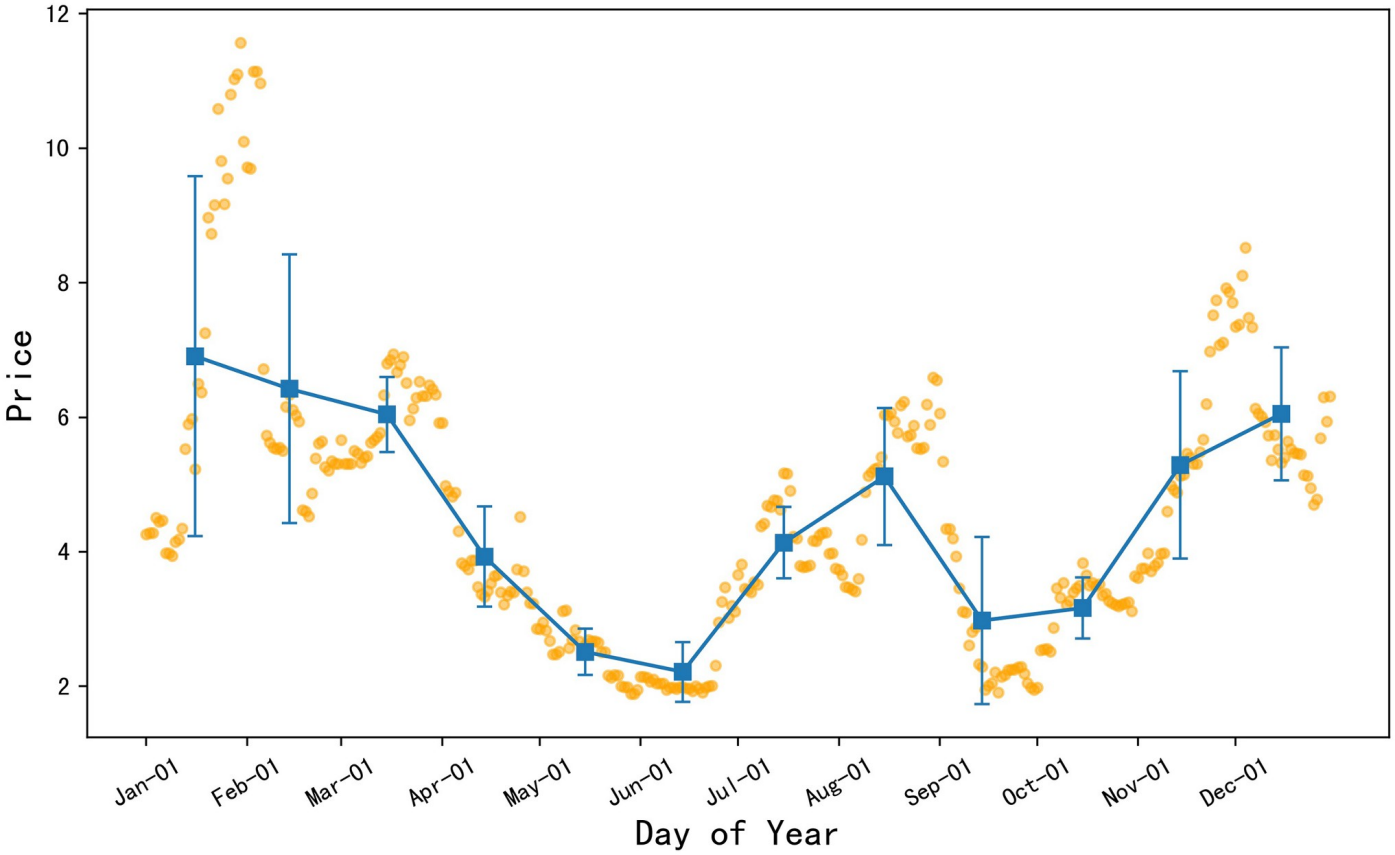
2022 daily average price chart for spiny cucumber.

According to Fig 5, the orange dots represent the daily price data of spiny cucumber in 2022, and the blue squares represent the monthly average price of spiny cucumber, with the error bars representing the standard deviation of monthly price data. The analysis reveals that the average monthly prices of spiny cucumber were relatively high in January, February, March, and December. However, starting from mid to late March, the price of spiny cucumber began to decline, reaching the lowest point of the year in June. Observing the price changes throughout the year, the prices of spiny cucumber in May and June were low and had smaller fluctuations, indicating stable prices in these two months. In contrast, the price volatility of spiny cucumber in January and February was more severe, with larger price changes.

#### In 2022, eggplant prices saw one peak and one trough

**Fig 6:**
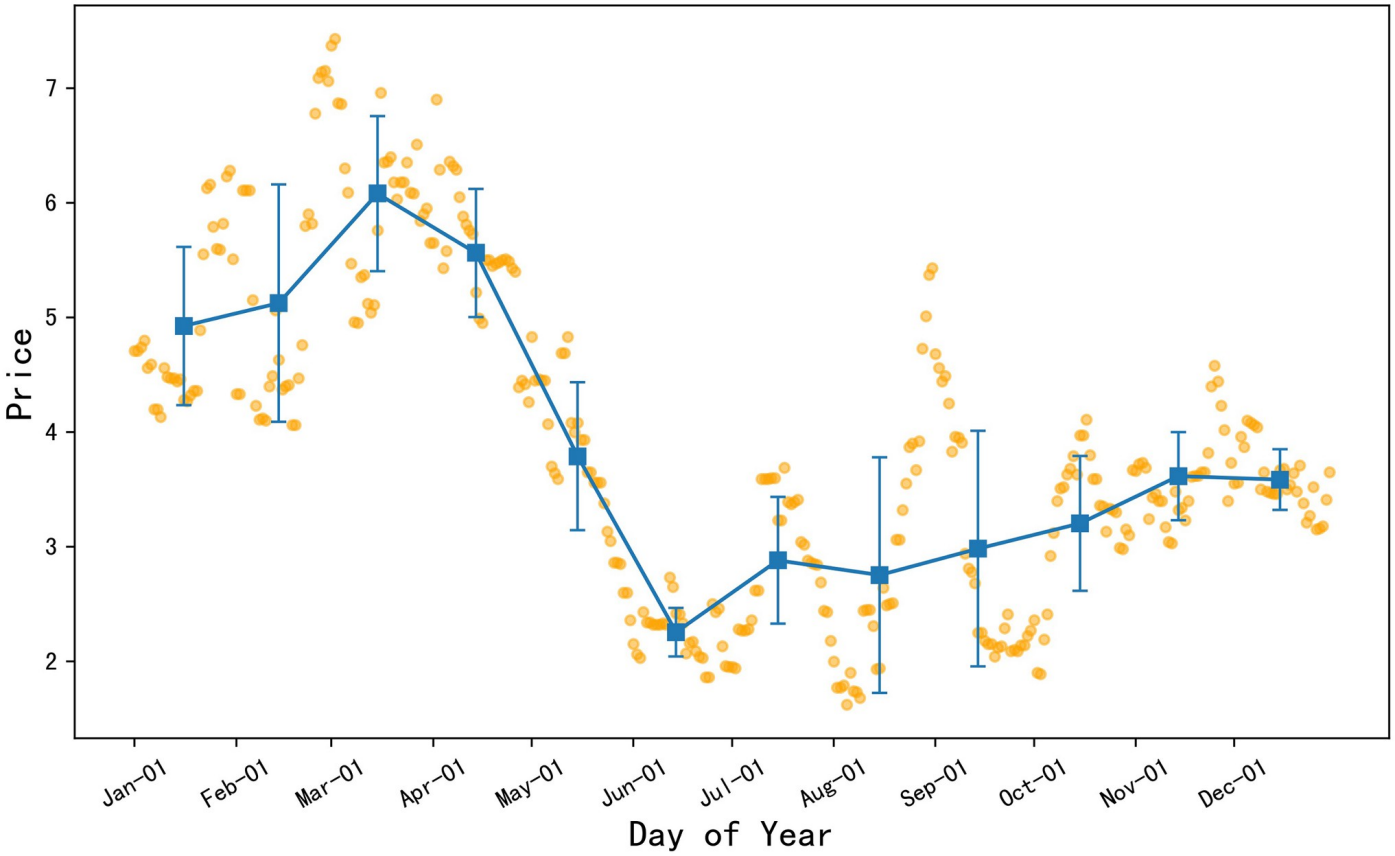
2022 daily average price chart for eggplant.

As shown in Fig 6, the orange dots represent the daily price data of eggplant in 2022, and the blue squares represent the monthly average price of eggplant, with error bars reflecting the standard deviation of the monthly price data. The trend for eggplant prices throughout the year shows that the average monthly prices were higher in January, February, March, and April. Starting from early April, the price of eggplant began to decline, reaching the lowest average price level of the year in June, and the volatility was also at its lowest, indicating the most stable prices. After late June, the price of eggplant began to rise again but remained significantly lower than the average prices of the first four months. Notably, the price fluctuations of eggplant were larger in August and September, with the highest values in the second half of the year occurring around the transition between these two months.

#### Carrot prices were relatively stable in autumn 2022

**Fig 7:**
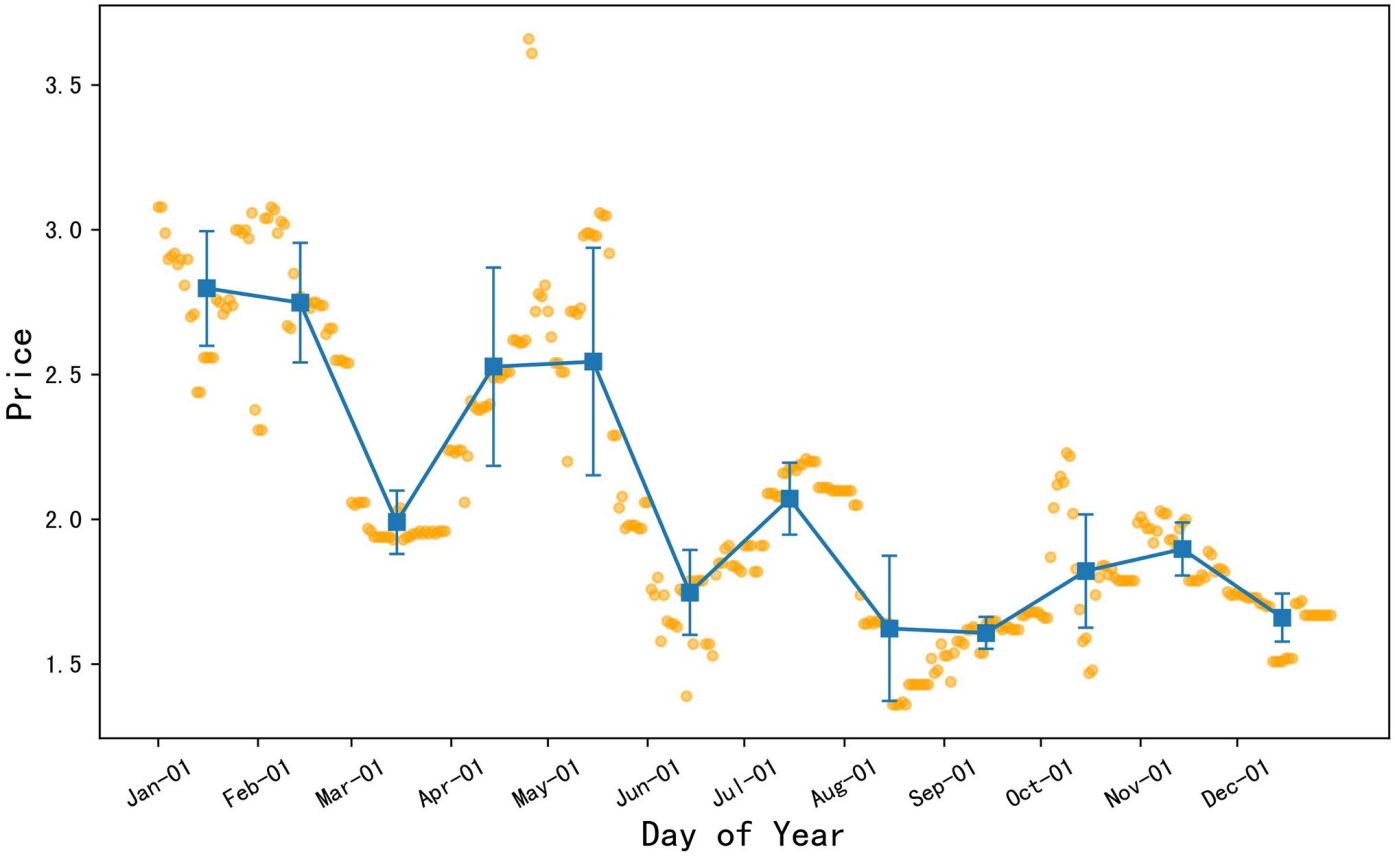
2022 daily average price chart for carrot.

As depicted in Fig 7, the orange dots represent the daily price data of carrots in 2022, and the blue squares signify the monthly average price of carrots, with error bars representing the standard deviation of the monthly price data. It can be deduced that the average monthly prices of carrots were at a higher level in January and February. The prices began to decline from late February and reached a small trough in March, which is a relative low point. However, after mid to late March, the prices began to rise again, reaching a small peak, or a relative high point, in April and May. Subsequently, carrot prices fell again, reaching another small trough in June, followed by an upward trend. Overall, the price trend of carrots showed a pattern of alternating peaks and troughs. Additionally, the prices of carrots were relatively stable in September, November, and December, with smaller fluctuations during these months.

#### There were two price peaks for potatoes in 2022

**Fig 8:**
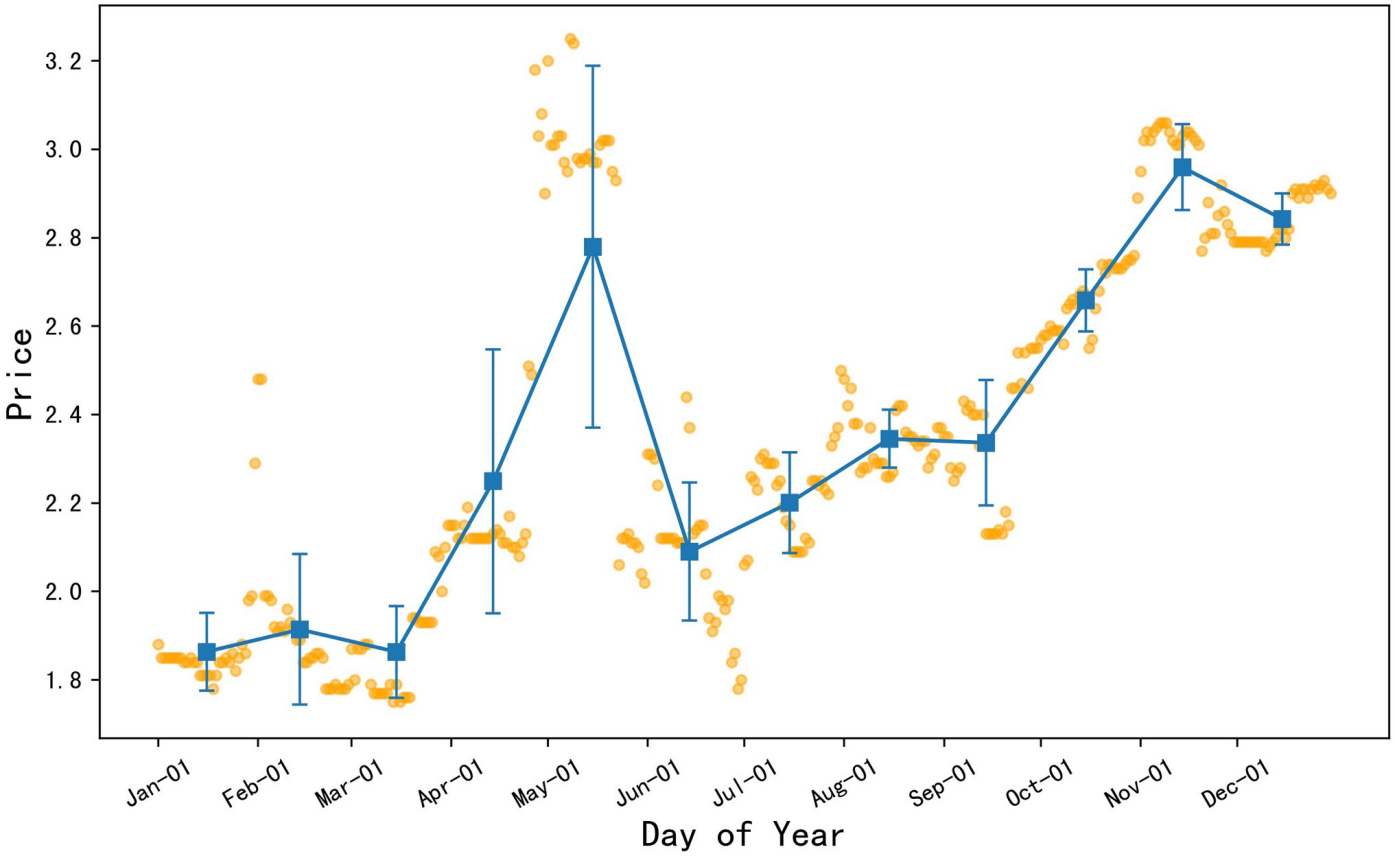
2022 daily average price chart for potato.

In accordance with the visual depiction of Fig 8, the orange dots represent the daily price data of potatoes in 2022, the blue squares represent the monthly average price of potatoes, and the error bars represent the standard deviation of the monthly price data. From the chart, it can be analyzed that the average prices of potatoes in January, February, and March were at a lower level throughout the year. However, starting from mid-March, the price of potatoes began to rise, reaching the highest value in the first half of the year in May, followed by a decline in prices. It’s worth noting that, at the end of June, the price of potatoes rose again and showed an overall upward trend in the second half of the year. Additionally, the price changes of potatoes were relatively stable in August, October, and December, while the price fluctuations were more dramatic in April and May.

#### The 2022 oyster mushroom prices showed an alternating trend of rises and falls

**Fig 9:**
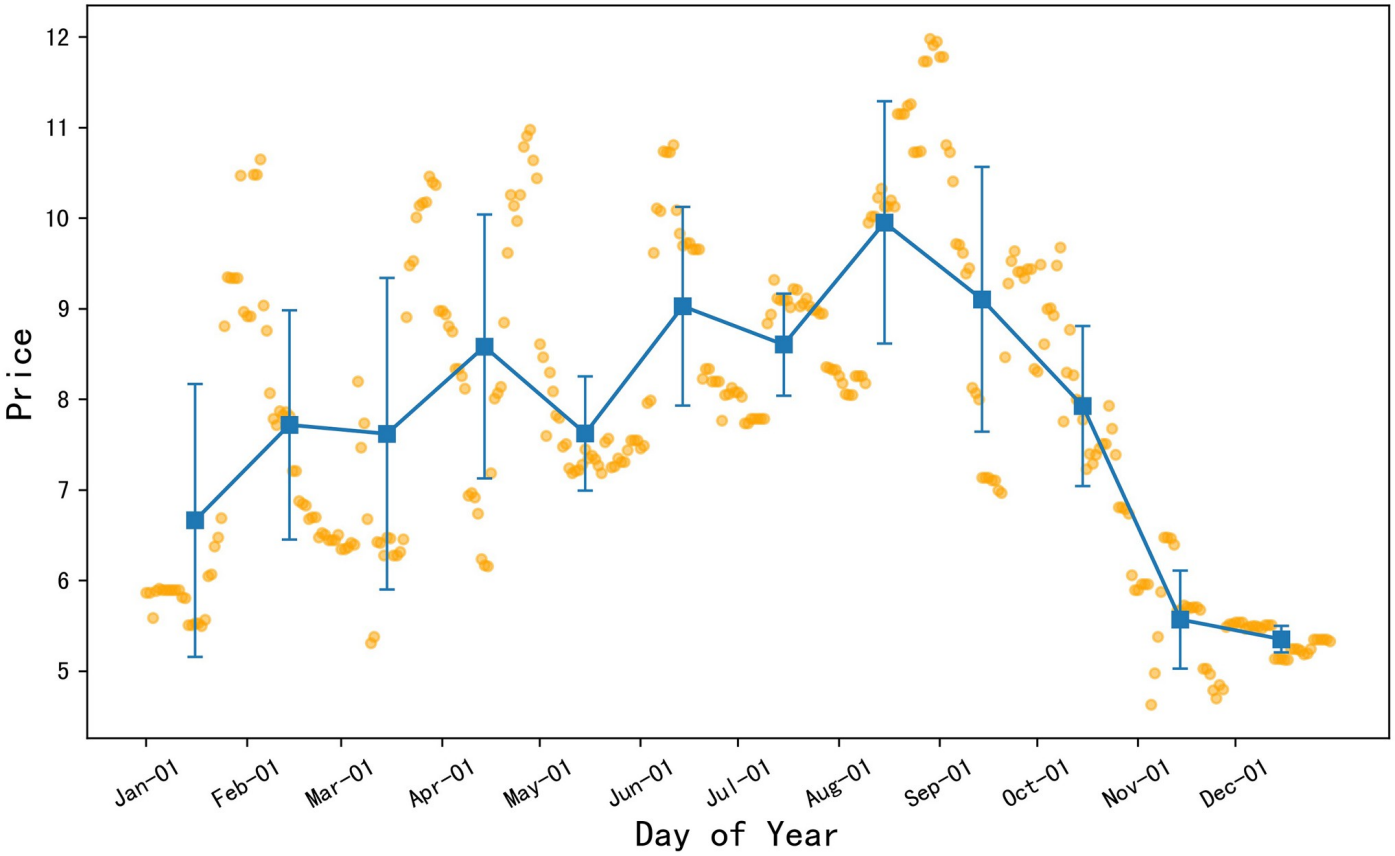
2022 daily average price chart for oyster mushroom.

Fig 9 shows that the orange dots represent the daily price data of oyster mushrooms in 2022, the blue squares represent the monthly average price of oyster mushrooms, and the error bars represent the standard deviation of the monthly price data. The analysis indicates that the average prices of oyster mushrooms were at a lower level in November and December, while the prices were relatively higher during the rest of the year. From January to October, the price fluctuations of oyster mushrooms were larger, indicating an instability in prices. Moreover, the basic price trend showed a pattern of monthly alternating rises and falls, meaning that during this period, the prices of oyster mushrooms exhibited a cyclical pattern of increases and decreases; particularly, the average price of oyster mushrooms in December was at the lowest level of the year and was also the most stable.

In summary, the following conclusions can be drawn: the annual price trends of the six vegetables show a strong non-linear tendency, with varying degrees of intra-monthly price volatility and no clear regularity. Furthermore, the price trends between different vegetable varieties do not exhibit consistent characteristics. Therefore, when using price data for forecasting, it is necessary to choose models with strong generalization ability and capable of fully capturing the characteristics of prior data, so as to more accurately predict future price trends.

## Model construction

LSTM (Long Short-Term Memory) is a type of recurrent neural network (RNN) architecture used for processing time series data; it addresses the issue of vanishing or exploding gradients that traditional RNNs often encounter when handling long-term dependencies by introducing a “gate” mechanism (Hochreiter, 1997; Graves, 2012; (Olah, 2015) [37–39].

Traditional neural network models consider only current input data without accounting for historical information, which makes them ill-suited for time series prediction tasks. In this study, Long Short-Term Memory (LSTM) model will be employed to forecast the trend of vegetable prices in Beijing’s wholesale markets for the upcoming week. Compared to traditional time series forecasting models, the LSTM model is capable of capturing long-term dependencies. It utilizes gate units to remember and selectively forget past information, thereby better handling of long-term dependencies in time series. This model can automatically learn features such as trends and seasonality in time series data without the need for manual differencing or seasonal decomposition. It demonstrates strong adaptive learning capabilities. Additionally, the model has higher robustness, tolerating noise and outliers to a certain extent, while also exhibiting good generalization across different time series datasets.

### Basic principles of LSTM algorithm

**Fig 10:**
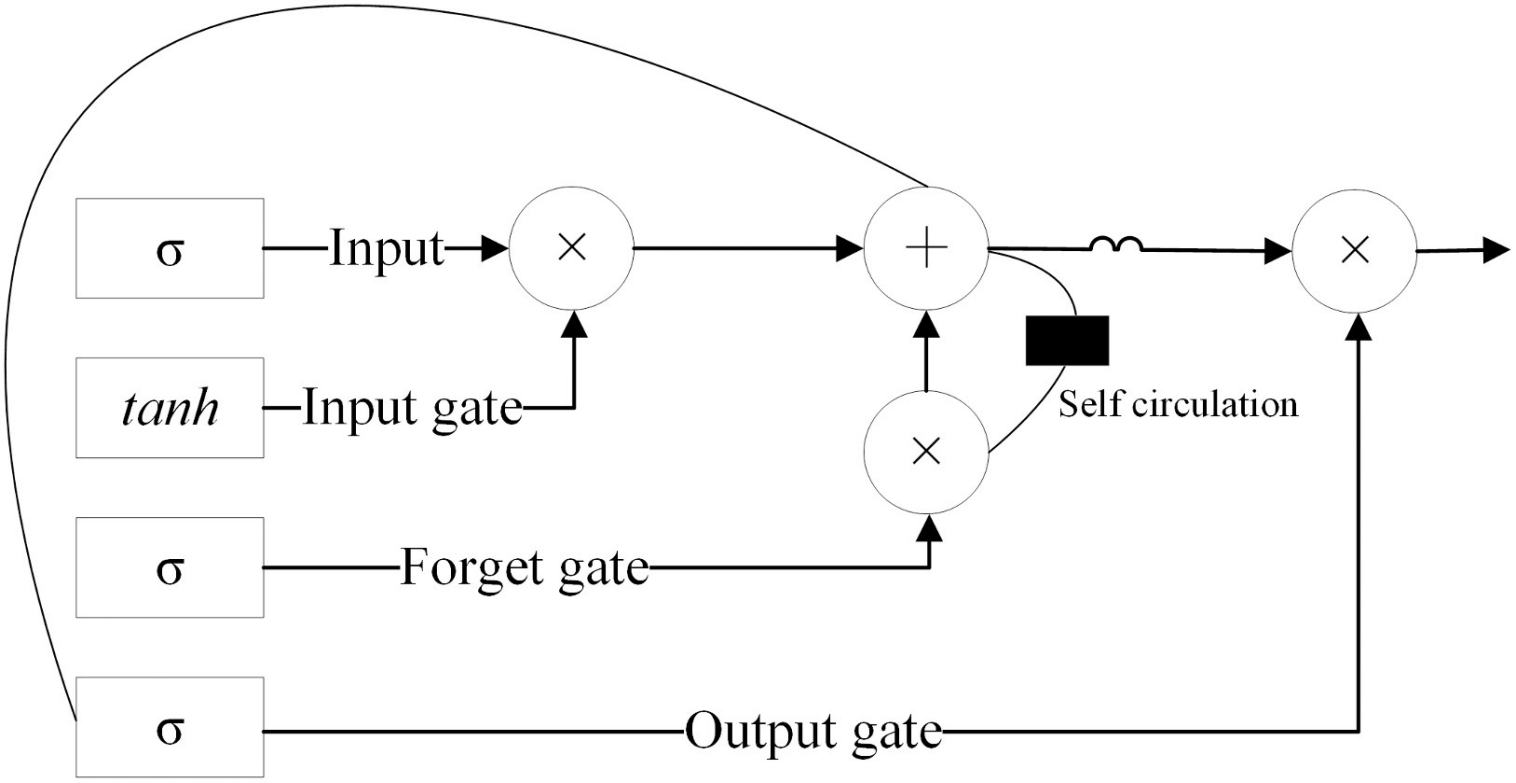
A schematic diagram of the LSTM network structure.

Basic Unit: LSTM contains a repeating module with three gates: the forget gate, the input gate, and the output gate. The network structure is illustrated as shown in the diagram below (Fig 10).

Forget gate: The forget gate determines what information from the previous cell state should be forgotten. It takes the previous hidden state (*H*_*t*−1_) and the current input (*X*_*t*_), then uses a sigmoid function (*σ*) to output a value between 0 and 1. This value is afterwards multiplied by the previous cell state (*C*_*t*−1_) to determine the information that is retained. The computation of the forget gate is shown in Eq (1).


$$f_{t}=\sigma \left(W_{f}\cdot \left[h_{t-1},x_{t}\right]+b_{f}\right)$$ (1)

Input gate: The input gate decides which positions of the cell state should be updated with the current input. It is composed of a sigmoid function and a tanh function. The sigmoid function outputs a vector between 0 and 1, which determines the extent of the update. However, the tanh function produces a vector with values ranging from -1 to 1, representing the new information to be stored. The computation of the input gate is represented by Eqs (2) and (3).


$$i_{t}=\sigma \left(W_{i}\cdot \left[h_{t-1},x_{t}\right]+b_{i}\right)$$ (2)

$$\tilde{C_{t}}=tanh\left(W_{c}\cdot \left[h_{t-1},x_{t}\right]+b_{c}\right)$$ (3)

Cell state Update: The cell state (*C*_*t*_) is updated by combining the results from the forget gate and the input gate. The forget gate has a direct impact on what old memories are to be retained, while the input gate dictates what new information should be added. This is how the long-term dependencies are updated and stored. The update of the cell state is calculated as shown in Eq (4).


$$C_{t}=f_{t}*C_{t-1}+i_{t}*\tilde{C_{t}}$$ (4)

Output gate: The output gate determines how the current hidden state should be updated based on the cell state. It is made up of a sigmoid function and a tanh function, which are used to produce a new hidden state. The computation of the output gate is shown in Eqs (5) and (6).


$$O_{t}=\sigma \left(W_{o}\cdot \left[h_{t-1},x_{t}\right]+b_{o}\right)$$ (5)

$$h_{t}=O_{t}*tanh\left(C_{t}\right)$$ (6)

Hidden state Output: At last, the current hidden state (*H*_*t*_) is computed based on the result of the output gate and the current cell state, and it is passed on to the next time step. The hidden state is the final output of the LSTM model.

The LSTM, through the aforementioned gating mechanisms, has found extensive application in areas such as time series forecasting, natural language processing, and speech recognition. It has become one of the formidable tools for managing sequential data.

### Determination of model parameters

For the construction of the relevant model, the Python programming language was employed. The software used in this study was PyCharm 2023.2 (Community Edition), version 17.0.7+7-b1000.6 x86_64, developed by JetBrains. After multiple training iterations, the best-performing set of hyperparameters was selected. The LSTM model’s structure comprises two layers, each with 32 neurons. The learning rate was set to 0.0027, and the dropout rate to 0.2 to prevent overfitting. The batch size was established at 500, with training iterations totaling 200 (Srivastava, 2014; Park, 2016) [40, 41]. The Adam optimizer was selected for optimizing the model’s performance. In contrast to other stochastic optimization algorithms, the Adam algorithm is more computationally efficient and has lower memory requirements, which is preferable for solving large-scale data problems (Zhang, 2015) [42].

During the training phase of each model, the first 80% of the dataset was allocated as the training set, and the remaining 20% served as the test set. Utilizing the trained LSTM model, the test set data was then employed to make daily price predictions for the upcoming week (from September 11, 2023, to September 17, 2023). The forecast results were visually presented.

### Metrics

The study employs four traditional ML measurements as performance evaluation metrics to evaluate the regarded approaches, including mean absolute error (MAE), root mean square error (RMSE), mean absolute percentage error (MAPE), and coefficient of determination (R^2^). They are respectively calculated according to formulas (7) to (10).


$$MAE=\frac{1}{n}\sum_{i=1}^{n}\left|y_{i}-{\hat{y}}_{i}\right|$$ (7)

$$RMSE=\sqrt{\frac{1}{n}\sum_{i=1}^{n}{\left(y_{i}-{\hat{y}}_{i}\right)}^{2}}$$ (8)

$$MAPE=\frac{1}{n}*\left(\sum_{i=1}^{n}\left|\frac{y_{i}-{\hat{y}}_{i}}{y_{i}}\right|\right)*100\%$$ (9)

$$R^{2}=1-\frac{\sum_{i=1}^{n}{\left(y_{i}-{\hat{y}}_{i}\right)}^{2}}{\sum_{i=1}^{n}{\left(y_{i}-\bar{y}\right)}^{2}}$$ (10)

**Fig 11:**
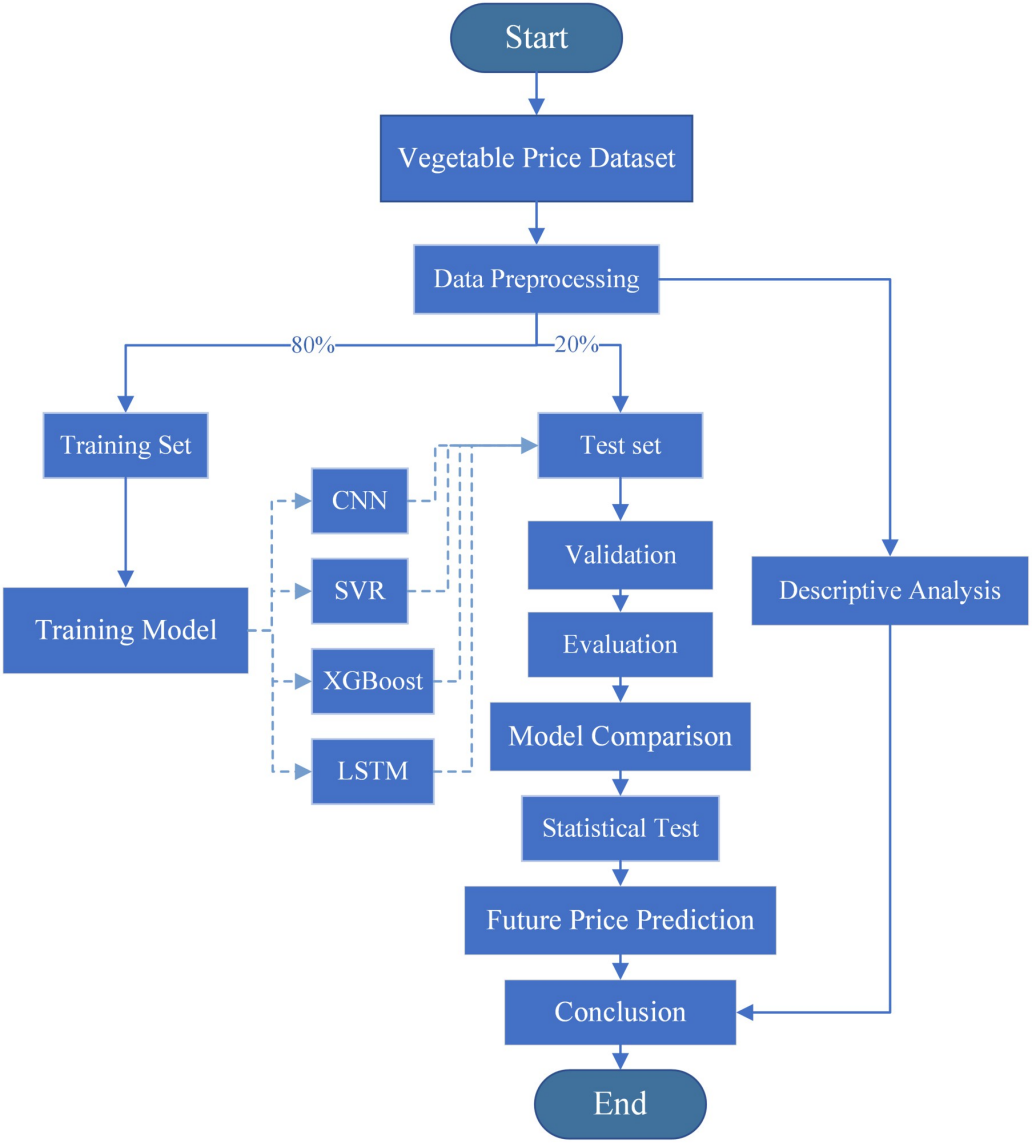
Flowchart of this study.

The process framework of the entire study is illustrated in Fig 11.

## Empirical results and analysis

### Goodness of fit for LSTM model

Prior to the LSTM model for short-term forecasting, an initial evaluation of its fitting adequacy on the test dataset is imperative. By observing its performance on the test set, we can judge and evaluate whether the LSTM model has learned the patterns and regularities within the data and if it can generalize well to unseen data. Such an analysis is instrumental in conducting a preliminary appraisal of the model’s prognostic efficacy.

#### Discussion on model loss function

**Fig 12:**
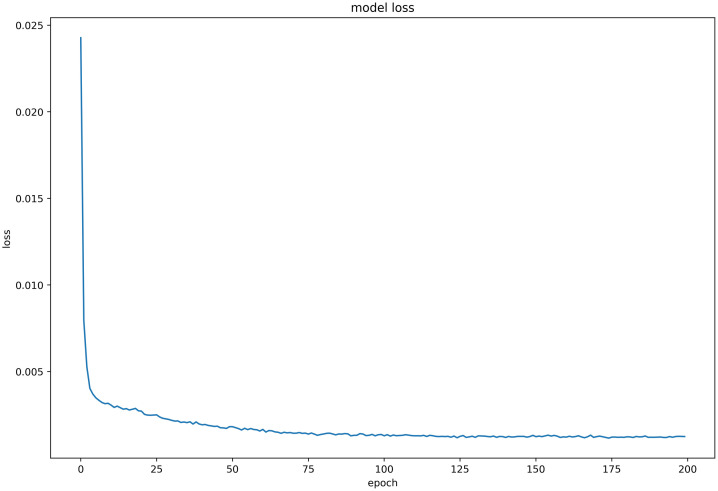
LSTM model loss function (Celery price data).

**Fig 13:**
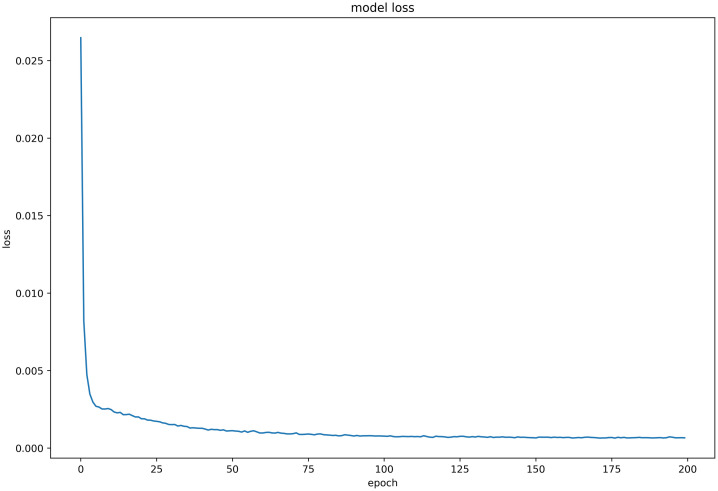
LSTM model loss function (Spiny cucumber price data).

**Fig 14:**
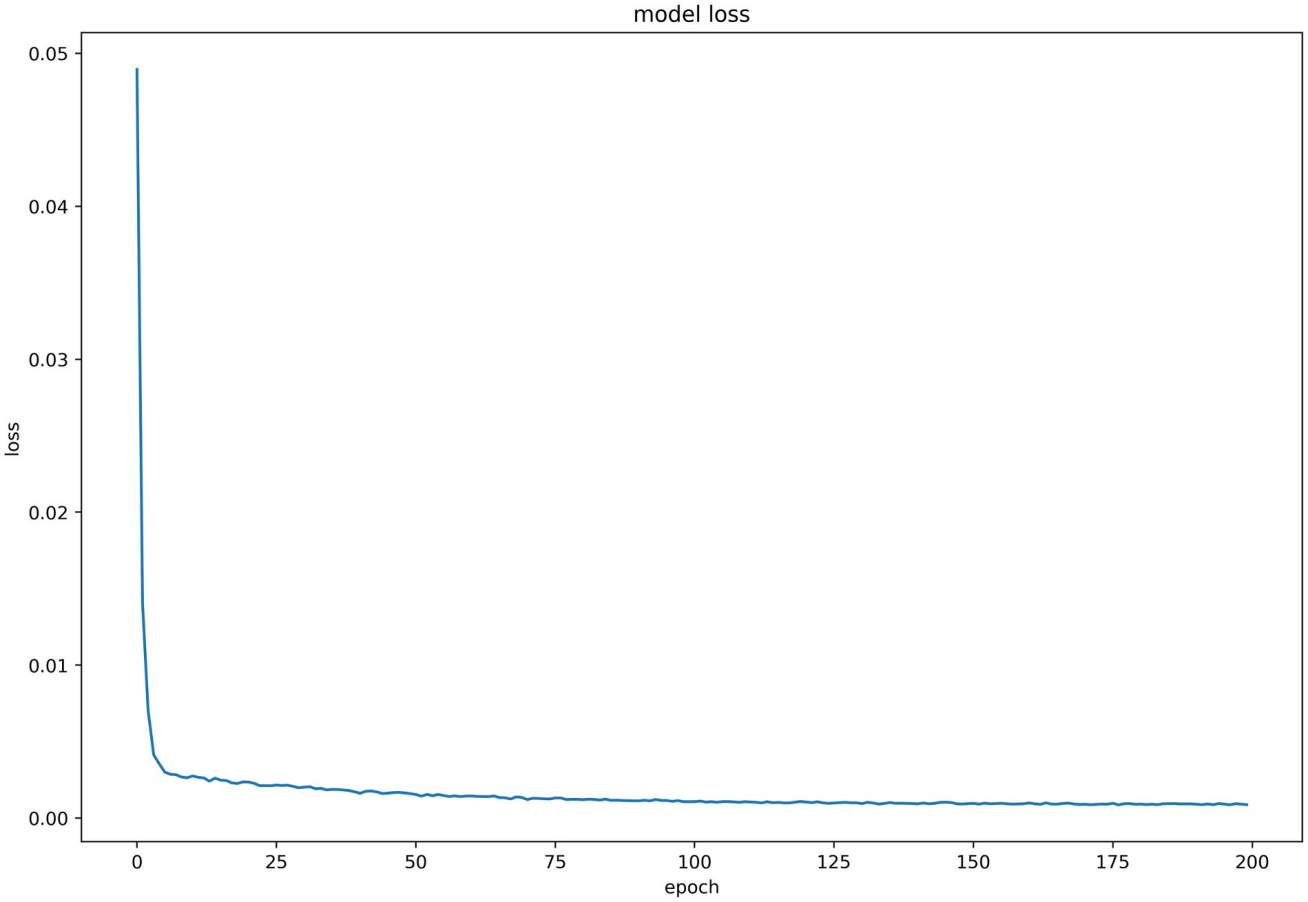
LSTM model loss function (eggplant price data).

**Fig 15:**
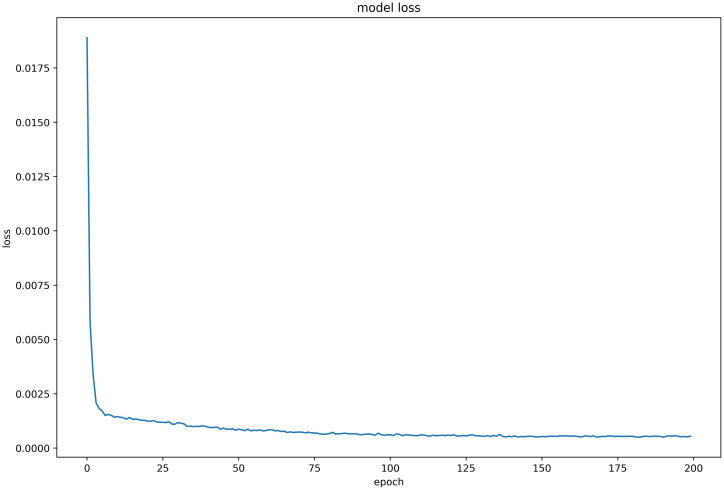
LSTM model loss function (Carrot price data).

**Fig 16:**
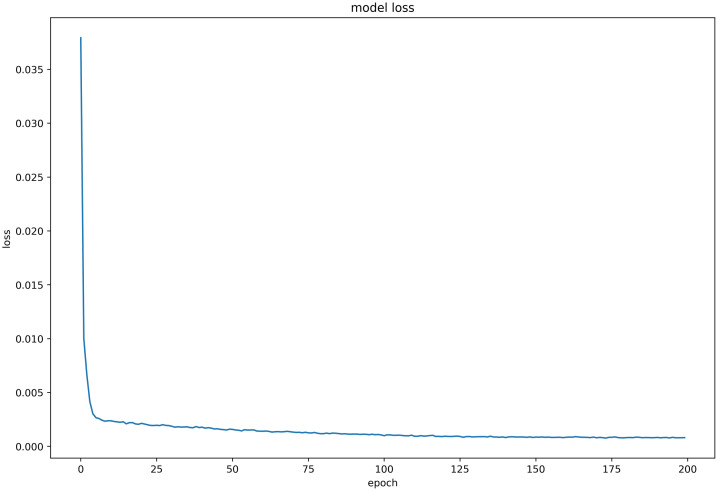
LSTM model loss function (Potato price data).

**Fig 17:**
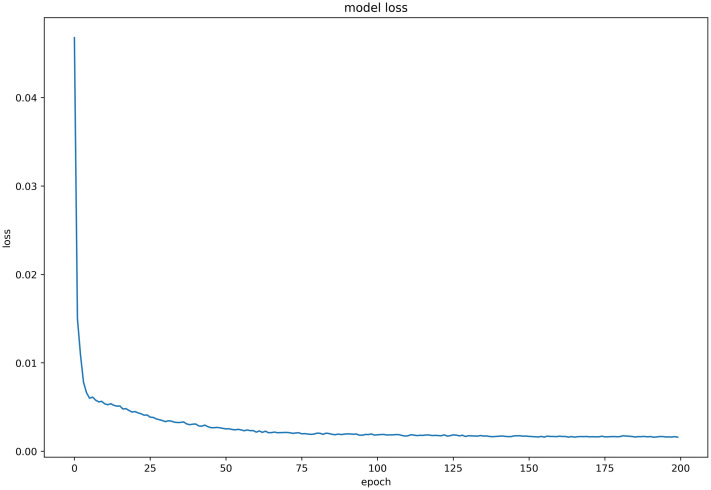
LSTM model loss function (Oyster price data).

Six LSTM models were trained using daily price data of celery, spiny cucumber, eggplant, carrot, potato, and oyster mushroom, and the results of their loss functions are shown in Figs 12–17 below. The results indicate that the loss function of the models trained with all six groups of data could decrease and stabilize within 25 iterations of training, achieving convergence; this suggests that the optimization algorithm of the model is capable of finding a parameter configuration close to the local optimal solution within a limited number of iterations. It also demonstrates the model’s fast adaptation and learning capabilities, which implies that using this model for future market price predictions is feasible.

#### Evaluation of model fitting effect

**Fig 18:**
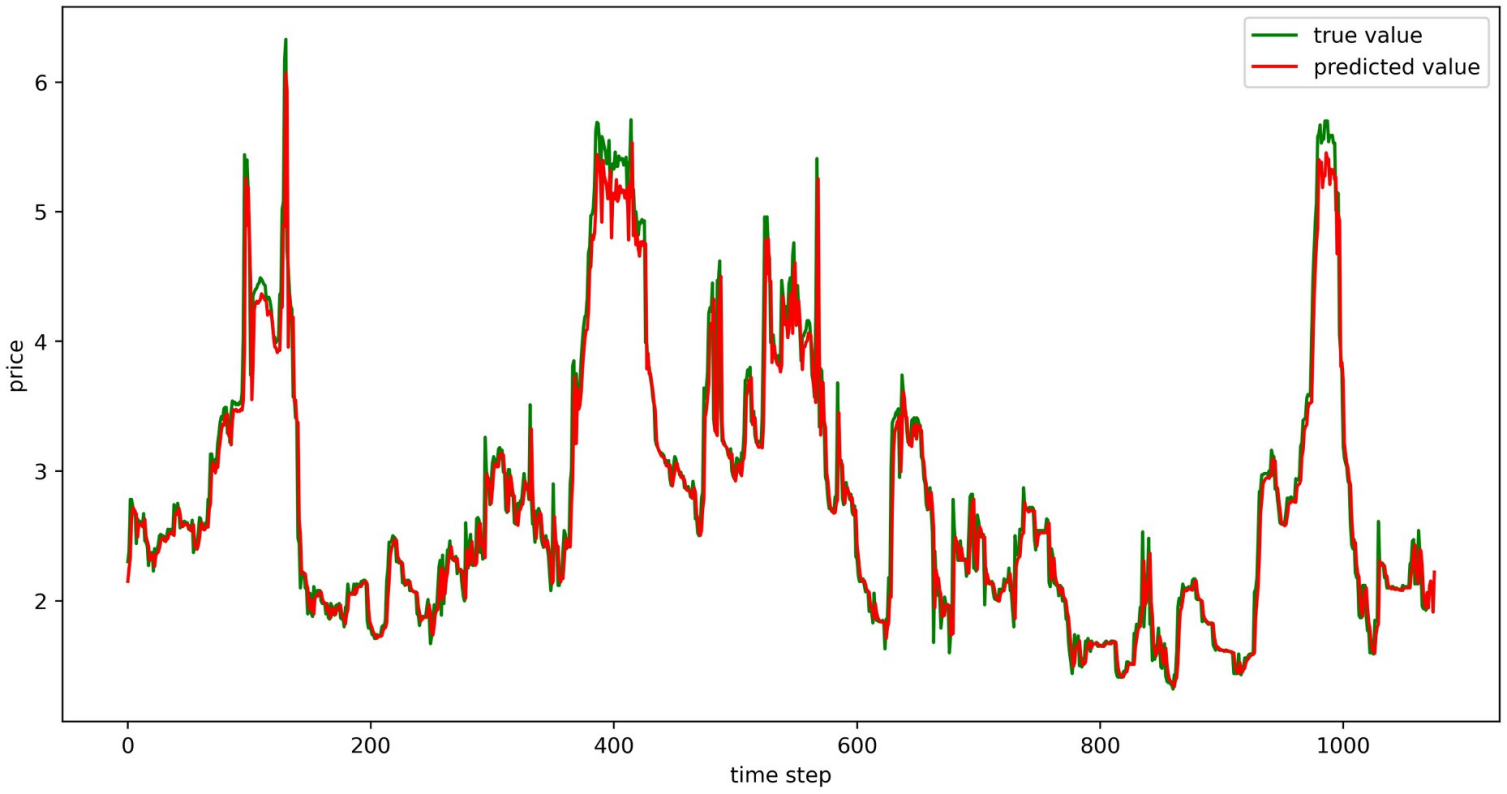
The fitting plot of LSTM model (Celery price data) on test set.

**Fig 19:**
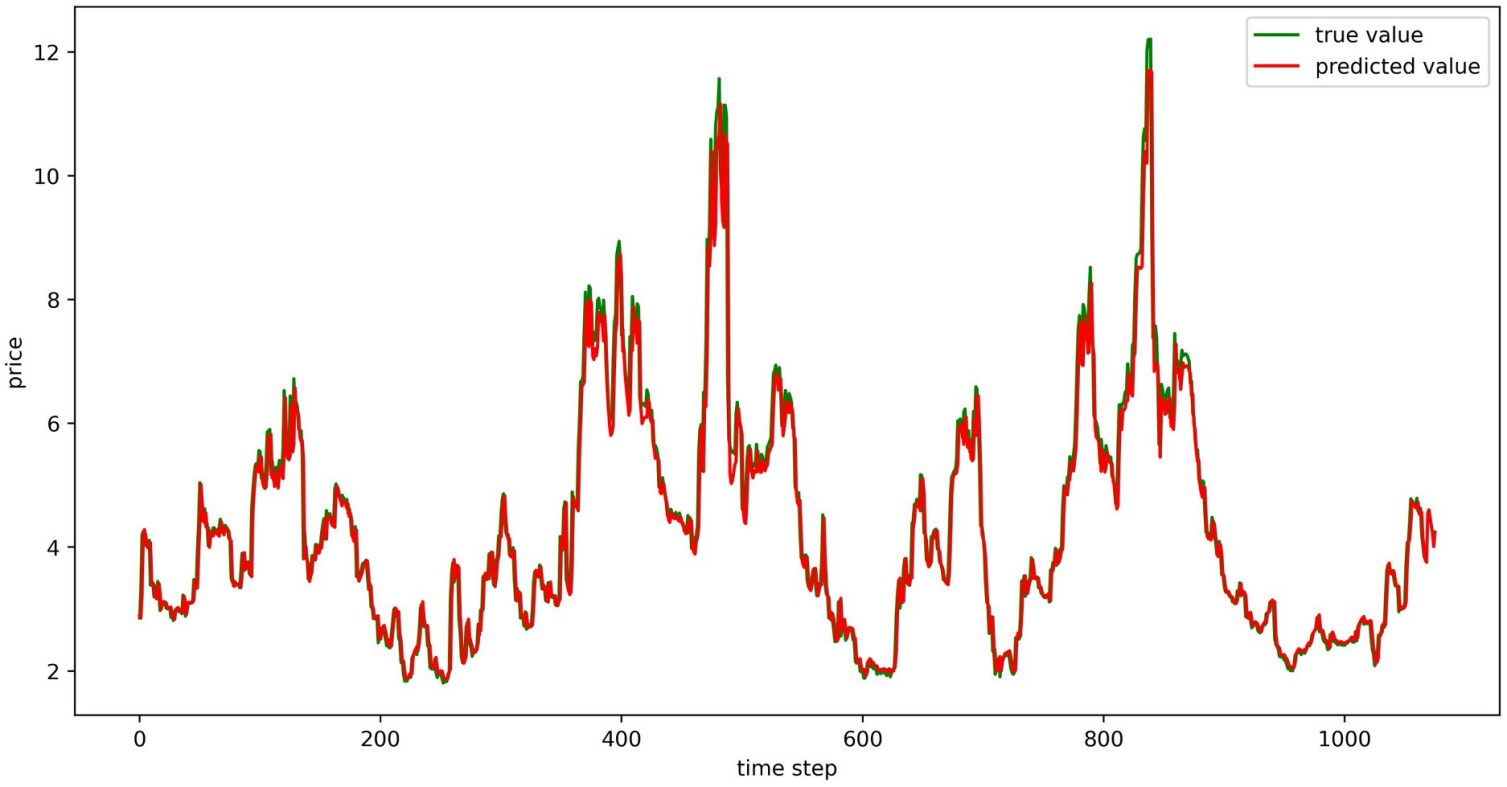
The fitting plot of LSTM model (Spiny cucumber price data) on test set.

**Fig 20:**
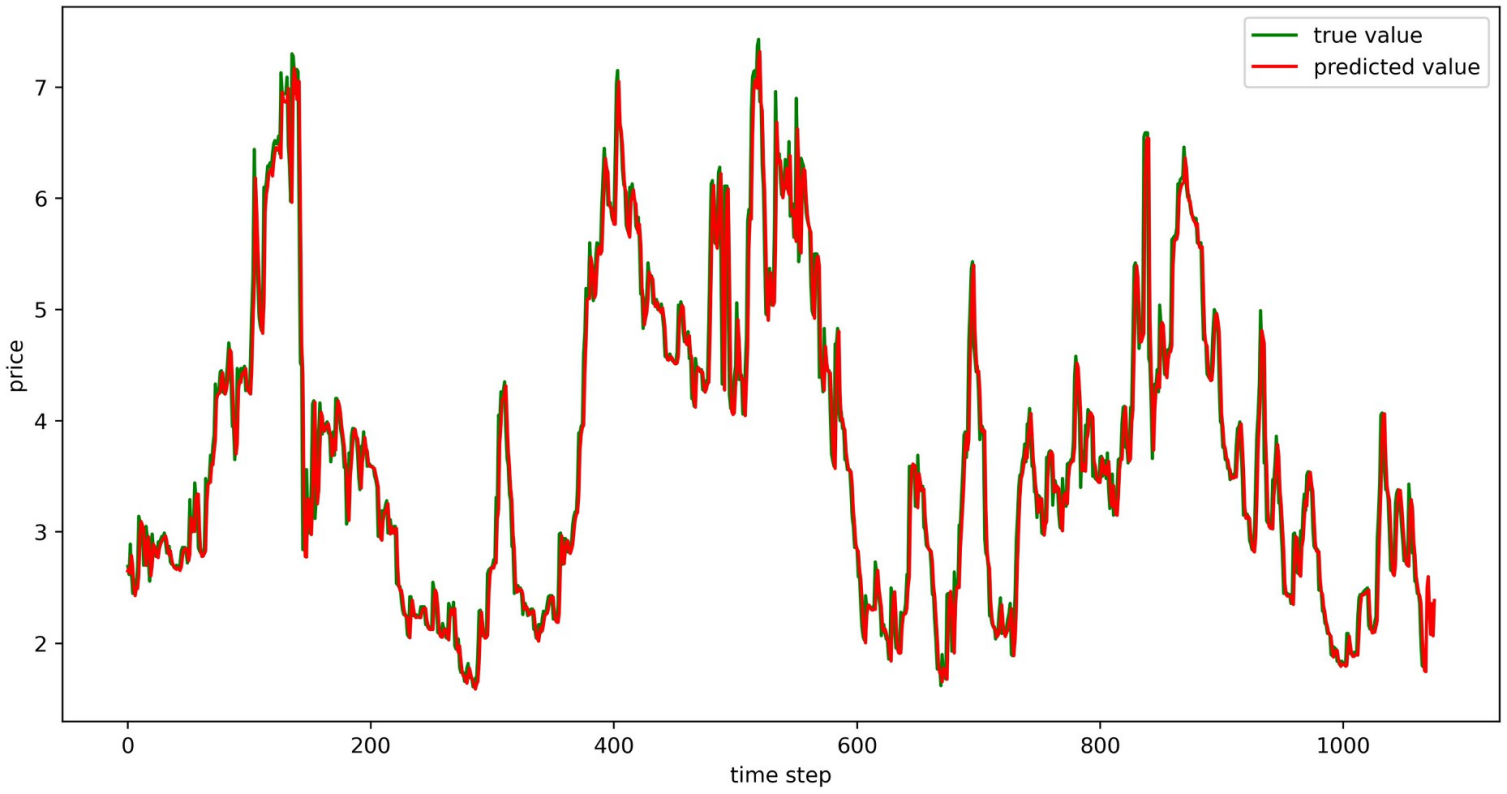
The fitting plot of LSTM model (Eggplant price data) on test set.

**Fig 21:**
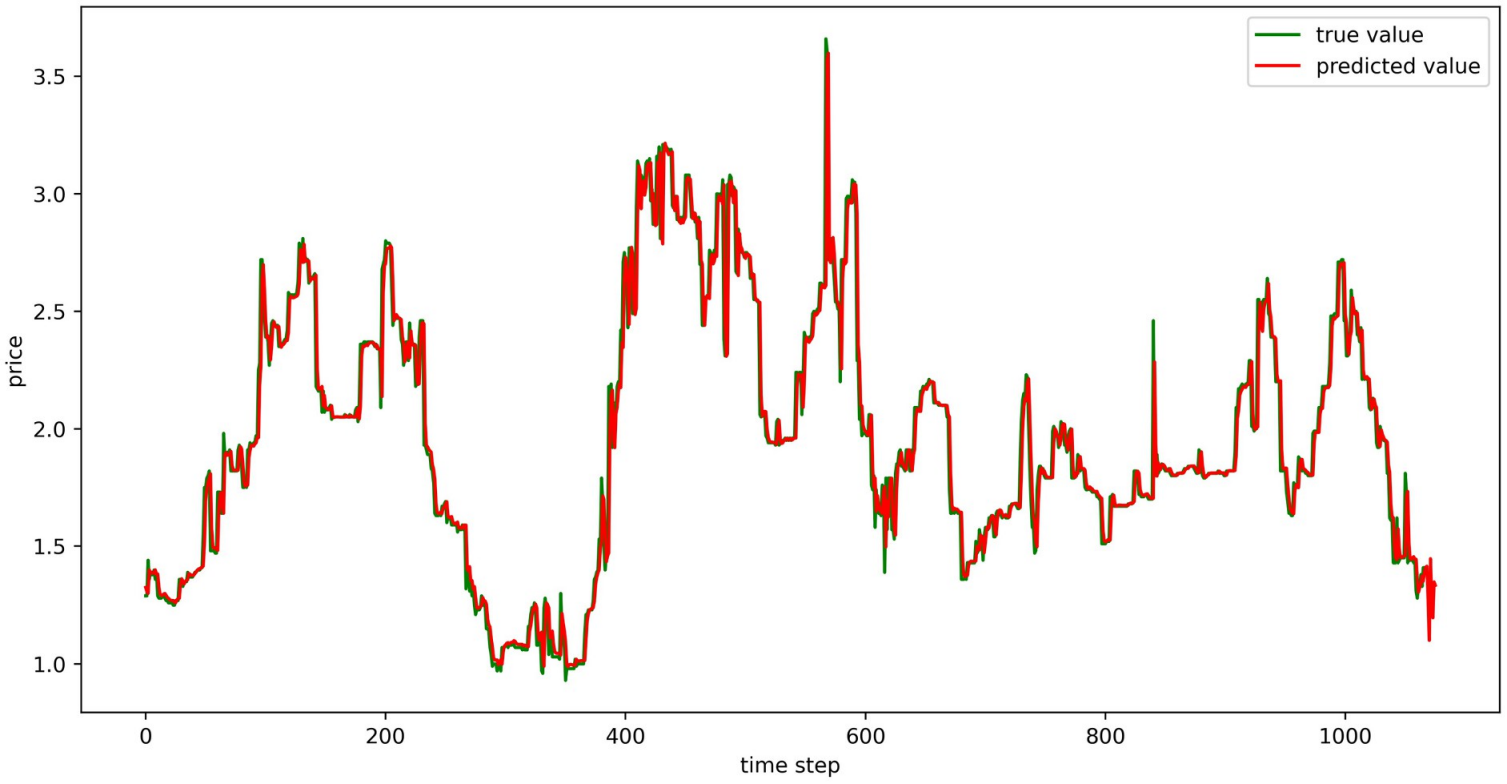
The fitting plot of LSTM model (Carrot price data) on test set.

**Fig 22:**
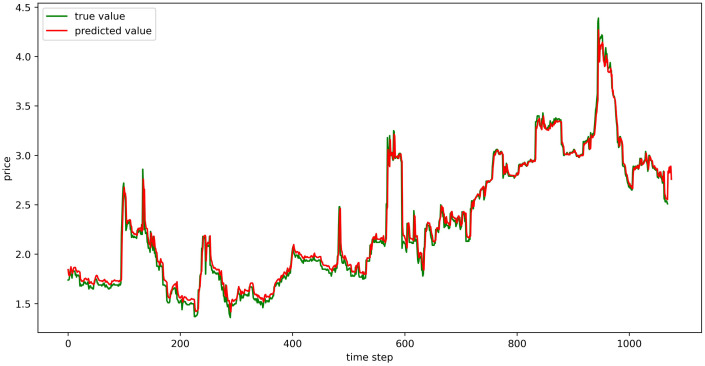
The fitting plot of LSTM model (Potato price data) on test set.

**Fig 23:**
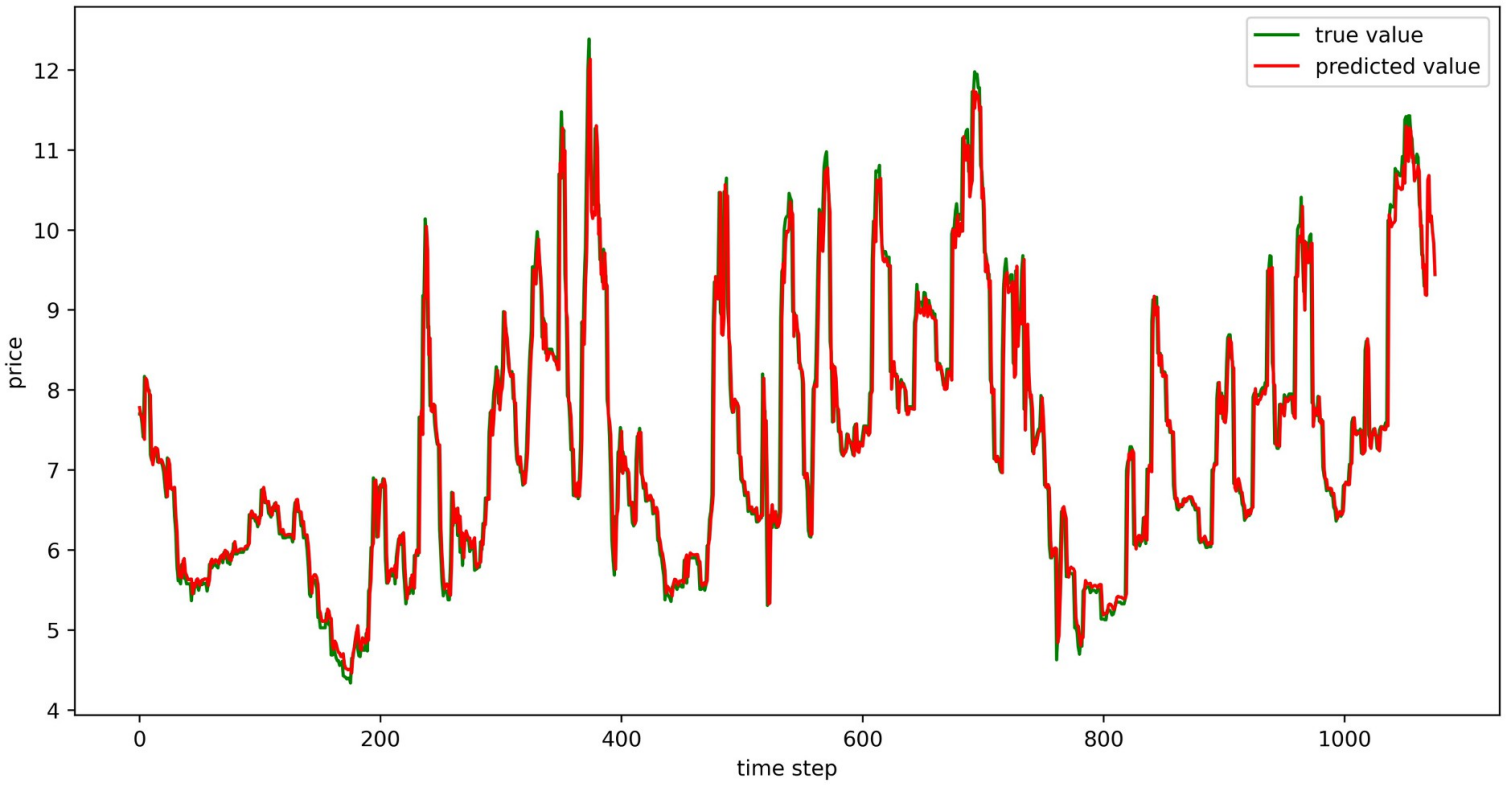
The fitting plot of LSTM model (Oyster mushroom price data) on test set.

The test set fitting images are shown in Figs 18–23, where the green lines represent the actual data and the red lines represent the predicted fit values obtained from the test set. The fitting images intuitively show that the LSTM model’s predictions are quite close to the actual values; the model’s predicted trend is also consistent with the actual data, showing good similarity. The model’s predictions exhibit reasonable volatility and key features without significant bias or error, and there are no obvious signs of overfitting or underfitting. It captures the relevant trends and periodicity of the test set data well. Overall, the LSTM model performs well on the six sets of data, which also indicates that the LSTM model has good generalization capabilities and can be used for subsequent predictions.

### Model prediction results

**Fig 24:**
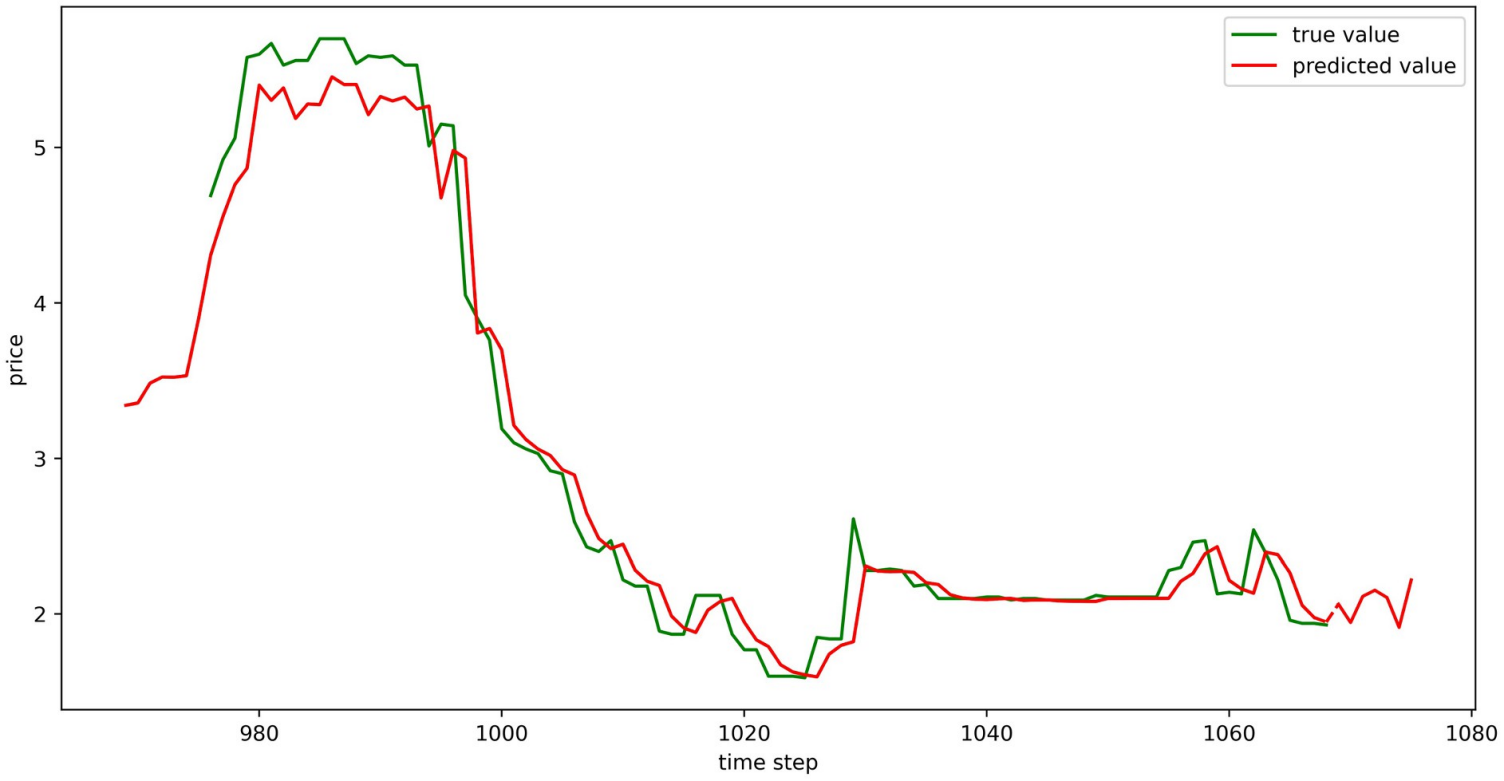
The prediction results of LSTM model (Celery price data).

**Fig 25:**
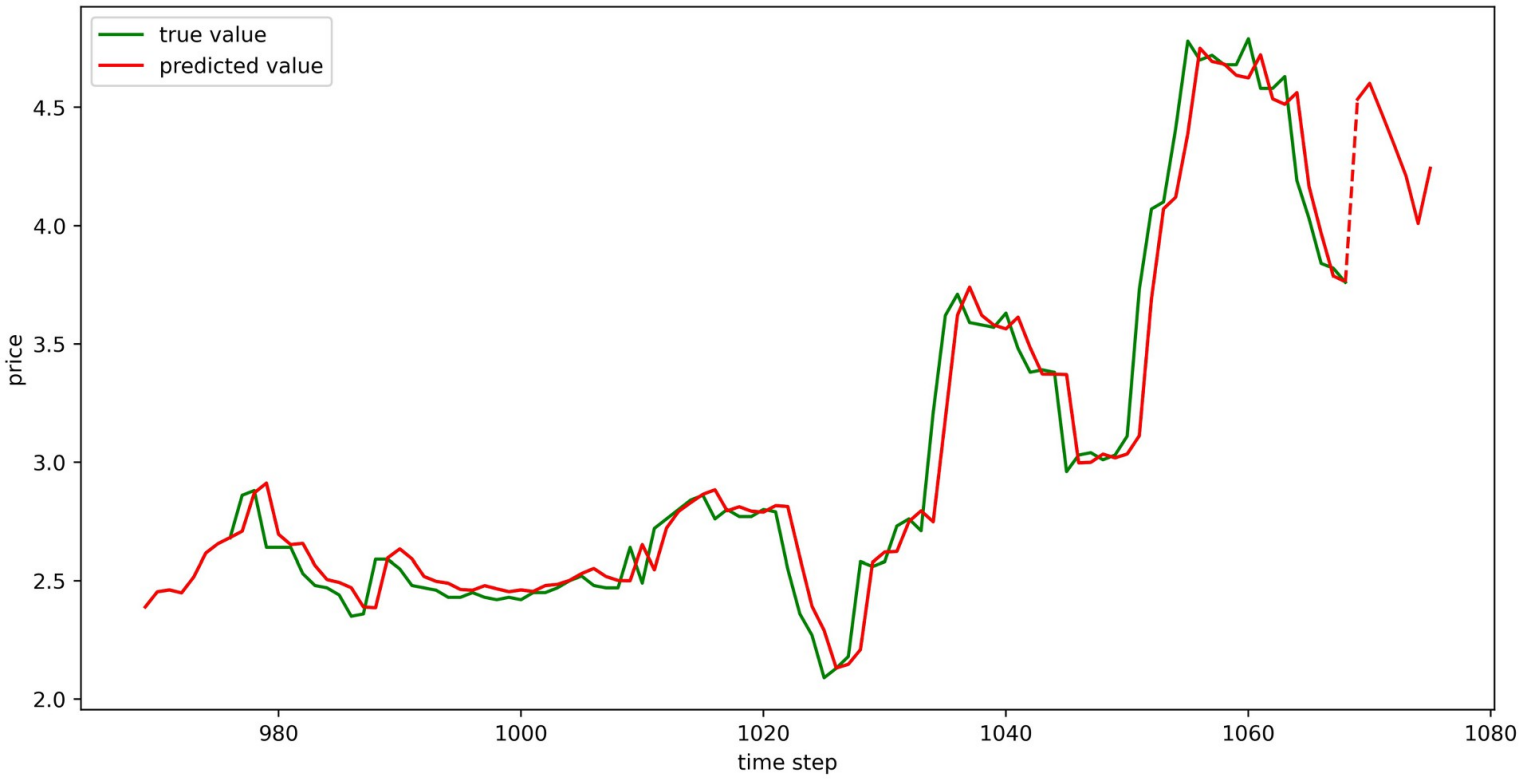
The prediction results of LSTM model (Spiny cucumber price data).

**Fig 26:**
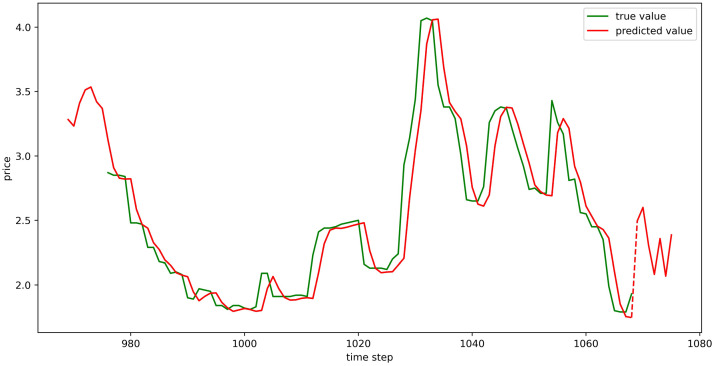
The prediction results of LSTM model (Eggplant price data).

**Fig 27:**
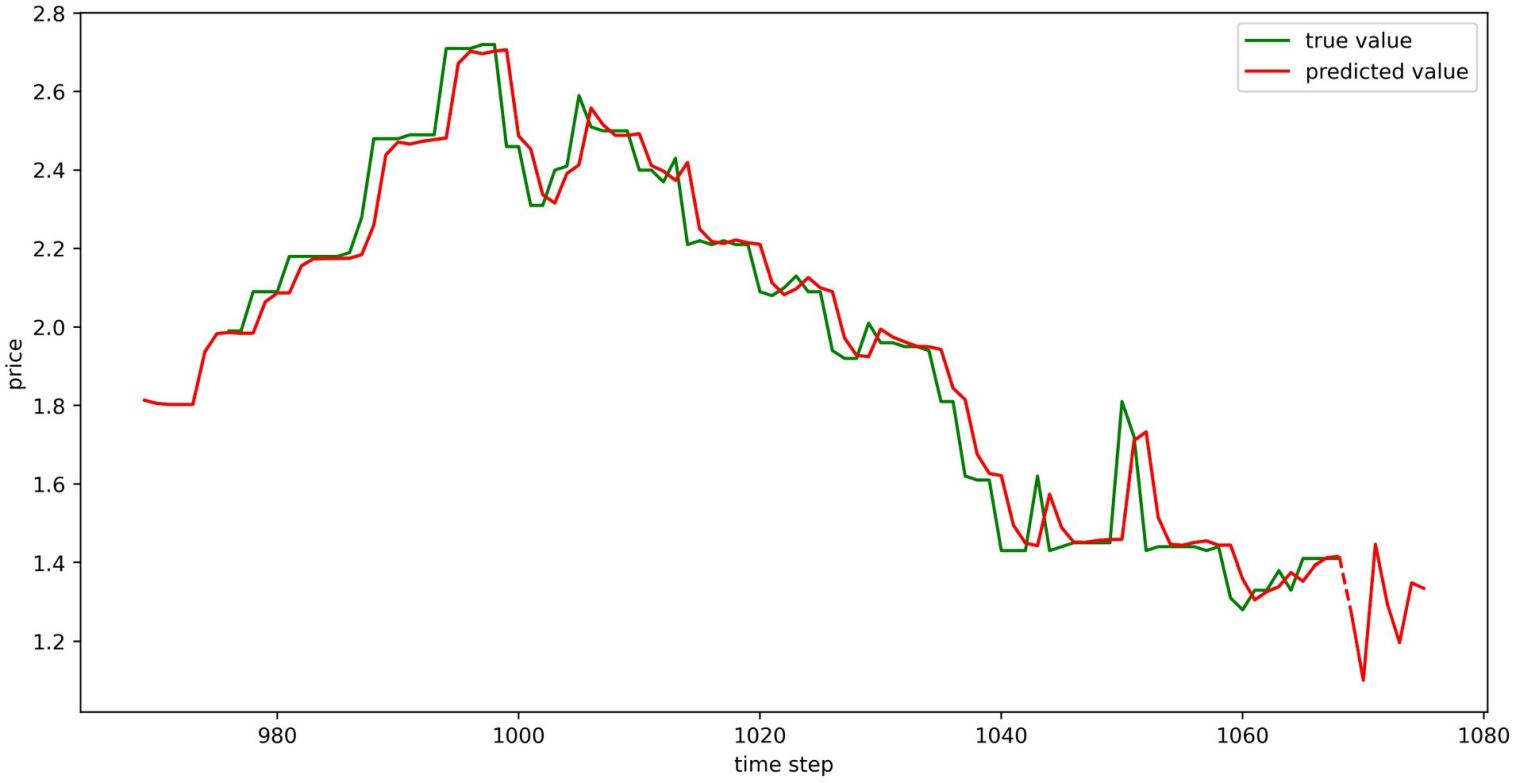
The prediction results of LSTM model (Carrot price data).

**Fig 28:**
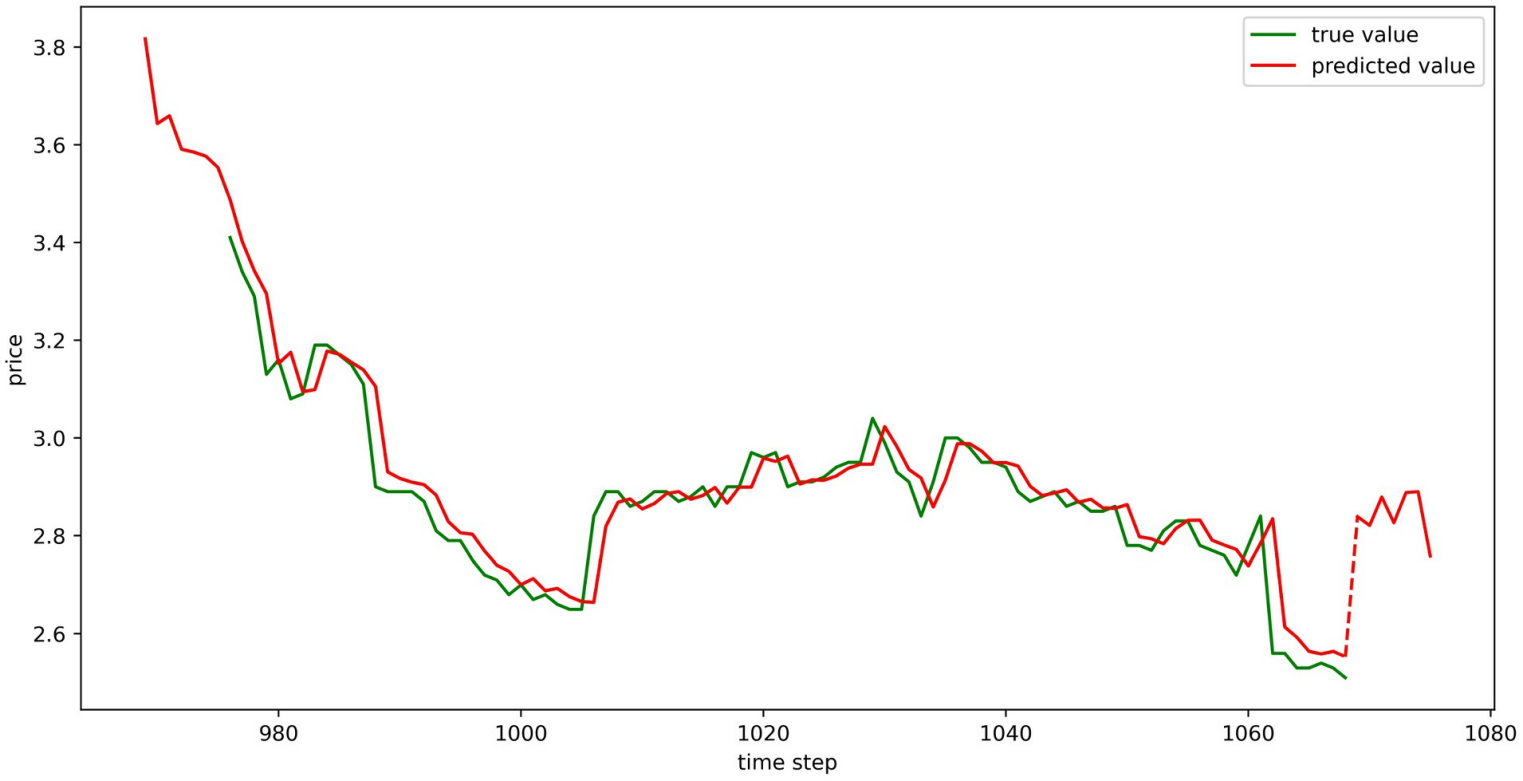
The prediction results of LSTM model (Potato price data).

**Fig 29:**
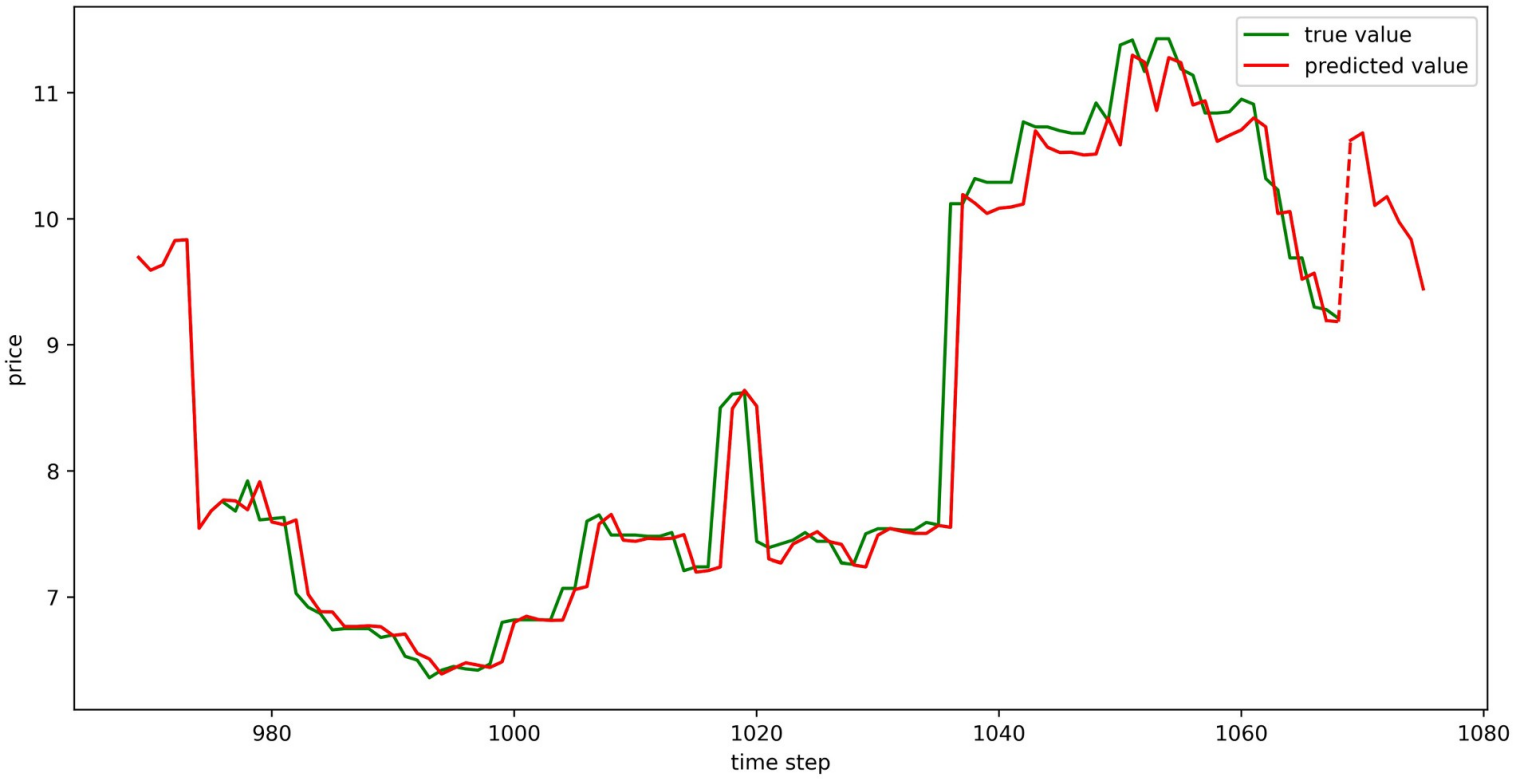
The prediction results of LSTM model (Oyster mushroom price data).

Using the aforementioned trained LSTM model, predictions were made for the prices of six types of vegetables for the upcoming week (September 11, 2023, to September 17, 2023), and the prediction results were visualized (Figs 24–29). In the images, the first 100 red line data points that overlap with the green lines represent the test set fitting results, and the red line after the dashed lines represents the forecasted values.

Using the trained model, we conducted a one-week price forecast. The forecast indicates that the price of celery will decrease initially, followed by a rise, and then it will maintain at a higher price point for a prolonged period. Spiny cucumber prices are forecasted to undergo an initial increase, a subsequent decrease, and then increase again, indicating a fluctuating trend over the next week. Eggplant prices are expected to demonstrate a “W” shaped movement, with one peak and two troughs observed within the week. Carrot prices are predicted to exhibit an alternating pattern of falling and rising prices. The price trajectory for potatoes is anticipated to form an “inverted W” within the week, characterized by two peaks and one trough. In contrast, oyster mushroom prices are projected to show a general downtrend with the occurrence of a slight peak during the week.

#### Evaluation of accuracy in prediction result

As indicated in Table 1, the forecast demonstrates that the model for the six groups of data accurately captures the volatility in the market prices of various vegetables. The comparison with actual market data shows that the model’s average accuracy surpasses 85%. Notably, the vegetables such as celery, carrots, oyster mushrooms, and spiny cucumbers have average accuracies above 90%, recorded at 93.3%, 92.9%, 90.2%, and 90.1% respectively. Among these, the lowest mean error rate is observed in celery at 6.7%, whereas potatoes present a higher mean error rate of 19.7%.

**Table 1:** LSTM model prediction results.

Varieties	Prediction Date	Predicted Value	Actual Value	Relative Error
**Celery**	2023-09-11	2.06	2.29	-10.0%
	2023-09-12	1.95	2.2	-11.4%
	2023-09-13	2.11	2.19	-3.7%
	2023-09-14	2.15	2.2	-2.3%
	2023-09-15	2.11	2.21	-4.5%
	2023-09-16	1.91	2.02	-5.4%
	2023-09-17	2.22	2.02	9.9%
**Spiny Cucumber**	2023-09-11	4.53	3.78	19.8%
	2023-09-12	4.6	3.96	16.2%
	2023-09-13	4.47	3.96	12.9%
	2023-09-14	4.34	3.94	10.2%
	2023-09-15	4.21	4	5.3%
	2023-09-16	4.01	4.12	-2.7%
	2023-09-17	4.24	4.14	2.4%
**Eggplant**	2023-09-11	2.5	2.09	19.6%
	2023-09-12	2.6	2.18	19.3%
	2023-09-13	2.3	2.01	14.4%
	2023-09-14	2.08	2.02	3.0%
	2023-09-15	2.36	2.03	16.3%
	2023-09-16	2.07	2.03	2.0%
	2023-09-17	2.39	2.04	17.2%
**Carrot**	2023-09-11	1.27	1.42	-10.6%
	2023-09-12	1.10	1.28	-14.1%
	2023-09-13	1.45	1.42	2.1%
	2023-09-14	1.29	1.42	-9.2%
	2023-09-15	1.20	1.34	-10.4%
	2023-09-16	1.35	1.32	2.3%
	2023-09-17	1.33	1.32	0.8%
**Potato**	2023-09-11	2.84	2.37	19.8%
	2023-09-12	2.82	2.36	19.5%
	2023-09-13	2.88	2.39	20.5%
	2023-09-14	2.83	2.37	19.4%
	2023-09-15	2.89	2.41	19.9%
	2023-09-16	2.89	2.39	20.9%
	2023-09-17	2.76	2.34	17.9%
**Oyster Mushroom**	2023-09-11	10.62	8.87	19.7%
	2023-09-12	10.68	8.98	18.9%
	2023-09-13	10.11	9.11	11.0%
	2023-09-14	10.18	9.11	11.7%
	2023-09-15	9.98	9.79	1.9%
	2023-09-16	9.84	9.77	0.7%
	2023-09-17	9.44	9.88	-4.5%

#### Evaluation of trend in prediction results

Within the context of the actual trading market, there is a pronounced preference for understanding the trends of future price movements over the absolute values derived from weighted average calculations. These trends are instrumental in aiding market participants with their decision-making processes. The following discussion will address the trend-based characteristics of the forecasted data.

**Fig 30:**
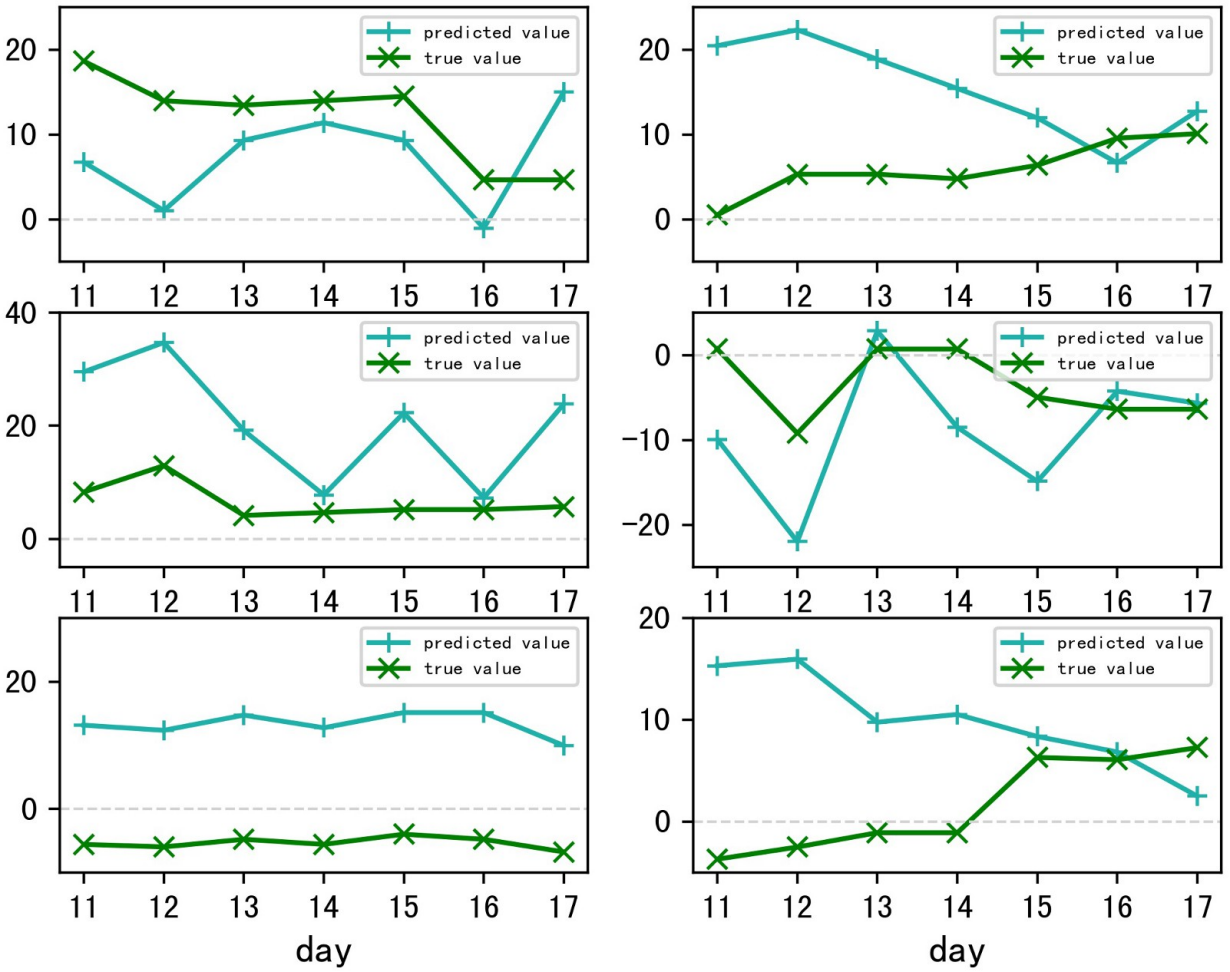
Trend chart of predicted and actual data.

The growth rates were determined by contrasting the forecasted figures against the actual prices on the 10th of September. For the vegetables such as celery, spiny cucumber, eggplant, and carrot, the concordance of predicted price variations with actual market trends exceeded 70%. This indicates that the majority of predictions, at least five out of seven, aligned with real market movements. Remarkably, spiny cucumber and eggplant predictions achieved a 100% match in trend direction, while the oyster mushrooms exhibited over 40% concordance. Despite the potato predictions not aligning as closely, the comparative analysis of seven-day sequential changes reflected a fundamental consistency with the actual trends (As shown in Fig 30). Thus, the model demonstrates commendable performance regarding the prediction of market trends.

#### Overall evaluation

The six vegetables analyzed in this study, namely celery, spiny cucumber, eggplant, carrot, potato, and oyster mushroom, represent four distinct categories: leafy, solanaceous, storage-resistant, and edible fungi, respectively. This diversity underscores the LSTM model’s strong generalization capabilities, permitting its application in forecasting market prices for various types of produce. The lower error rate further reflects the model’s forecasting precision and its interpretative strength regarding market dynamics. By leveraging this model, stakeholders can base their market strategies on robust data, facilitating more accurate decision-making, timely market trend adaptation, and intelligent trade or investment planning. This approach aims to reduce risk exposure while enhancing potential financial gains.

### Comparison with other models

To underscore the superior performance of the LSTM model, we conducted a comparative analysis against state-of-the-art traditional machine learning techniques. We applied these models to an identical dataset for a congruent predictive task–forecasting vegetable prices in Beijing.

For this purpose, Convolutional Neural Network (CNN) architecture was enlisted, utilizing the adam optimizer with a learning rate meticulously set to 0.001. Additionally, we included Support Vector Regression (SVR) and XGBoost in our comparative study, which were deployed in their default configurations as provided by the scikit-learn library, without any fine-tuning.

The comparison results are detailed in Table 2, where the LSTM model and the CNN model are shown as the average of each performance metric over 10 independent experiments, respectively. The outcomes clearly illustrate the LSTM’s adeptness at handling the intricacies of the specified prediction challenge. The LSTM model not only adapted well to the task but also demonstrated a marked improvement in predictive accuracy over the alternative models that were evaluated.

**Table 2:** Comparison of results between traditional ML methods and LSTM.

Varieties	Method	R2	MAE	RMSE	MAPE
**Celery**	LSTM	0.953	0.131	0.226	0.044
	CNN	0.873	0.225	0.370	0.072
	XGBoost	-0.635	1.005	1.757	0.365
	SVR	-1.023	1.098	2.174	0.334
**Spiny Cucumber**	LSTM	0.962	0.215	0.366	0.047
	CNN	0.915	0.346	0.549	0.078
	XGBoost	0.407	1.074	2.105	0.251
	SVR	-0.279	1.524	4.541	0.313
**Eggplant**	LSTM	0.952	0.186	0.302	0.050
	CNN	0.894	0.314	0.446	0.086
	XGBoost	-0.059	1.055	1.987	0.262
	SVR	-0.363	1.189	2.557	0.292
**Carrot**	LSTM	0.956	0.055	0.113	0.028
	CNN	0.904	0.108	0.166	0.055
	XGBoost	-1.253	0.651	0.646	0.336
	SVR	-1.017	0.611	0.578	0.279
**Potato**	LSTM	0.982	0.052	0.086	0.024
	CNN	0.961	0.081	0.126	0.034
	XGBoost	-0.478	0.613	0.602	0.288
	SVR	-0.373	0.564	0.559	0.217
**Mushroom**	LSTM	0.941	0.221	0.405	0.029
	CNN	0.869	0.369	0.601	0.048
	XGBoost	0.236	1.166	2.106	0.168
	SVR	-0.983	1.819	5.462	0.22

### Statistical test

To confirm the statistical significance of the obtained results, it’s essential to conduct a validation. This involves taking the optimal results from each of the 10 runs for all types of vegetables and methodologies, and considering them as individual data sets. To determine whether to use a parametric test or a non-parametric test, the assumption of normality has been assessed using the Shapiro-Wilk test for normality, calculating separate p-values for each method in question. Since all the p-values obtained are below the extremely low threshold of 1.475e-14, the null hypothesis (H0) can be confidently dismissed at the 0.05 significance level. This leads to the conclusion that the distribution of the simulation results is not normal. The Shapiro-Wilk test results are provided in Table 3, where *** indicates a significance level of less than 0.001.

**Table 3:** Shapiro-Wilk test statistic.

Varieties	LSTM	CNN	SVR	XGBoost
**celery**	0.908***	0.935***	0.951***	0.906***
**spiny cucumber**	0.913***	0.918***	0.923***	0.968***
**eggplant**	0.949***	0.948***	0.947***	0.884***
**carrot**	0.981***	0.978***	0.891***	0.808***
**potato**	0.929***	0.928***	0.949***	0.888***
**oyster mushroom**	0.956***	0.958***	0.933***	0.917***

*Note*: *w* statistics in parentheses

* *p* < 0.1,

** *p* < 0.05,

*** *p* < 0.01

Given that the Shapiro-Wilk test indicated that the data does not conform to a normal distribution, precluding the use of parametric tests, the alternative non-parametric approach is necessary. Hence, the Wilcoxon signed-rank test was employed, utilizing the same collection of data series that contain the best outcomes from each run. The LSTM model served as the control algorithm. The collected findings are compiled in Table 4. For the trials involving LSTM, the p-values obtained were most of below the significance threshold of 0.05. From this, it can be inferred that the LSTM technique demonstrates a statistically significant improvement over its competing methods.

**Table 4:** P-values from the Wilcoxon signed-rank test.

Varieties	LSTM	CNN	SVR	XGBoost
**celery**	N/A	4.796e-0.5	<2.2e-16	0.0184
**spiny cucumber**	N/A	<2.2e-16	<2.2e-16	0.434
**eggplant**	N/A	<2.2e-16	<2.2e-16	<2.2e-16
**carrot**	N/A	<2.2e-16	<2.2e-16	<2.2e-16
**potato**	N/A	<2.2e-16	<2.2e-16	0.0008
**oyster mushroom**	N/A	<2.2e-16	<2.2e-16	<2.2e-16

## Conclusions and discussions

### Conclusions

The yearly price trajectory of vegetable prices in Beijing’s market is characterized by pronounced non-linearity, with monthly price volatility showing no distinct regular patterns. Furthermore, the price trends across various vegetable categories types lack significant consistency.

The six vegetables selected in this article belong to four different categories: leafy vegetables, solanaceous vegetables, storage-resistant vegetables, and edible fungi. The predictive accuracy and fitting performance of LSTM model surpass those of other traditional machine learning models. This also suggests that the model can be generalized and applied to the price prediction of a broader range of vegetable categories in Beijing, demonstrating LSTM model’s good generalization in handling nonlinear and irregular data.

The forecast results reveal distinct fluctuation characteristics for the different categories of vegetables. In the coming week, celery prices initially increase and subsequently decline over the coming week Conversely, spiny cucumber prices are projected to undergo an initial rise, followed by a decline and a secondary increase. Eggplant prices are expected to display a “W” shaped trend, while carrot prices may exhibit alternating rising and falling patterns. Potato prices are foreseen to trace an inverse “W” trajectory, and oyster mushroom prices are predicted to trend downwards.

The model’s forecasted outputs boast an average accuracy rate exceeding 85%, with the directional trends of these predictions aligning closely with real-world observations, underscoring the model’s robust predictive precision. Thus, LSTM model possesses commendable predictive accuracy, effectiveness, and reliability in practical forecasting scenarios.

### Discussions

In the previously mentioned study, although LSTM model demonstrated an effective capability to predict the daily average vegetable prices across seven major wholesale markets in Beijing, the research still has certain limitations. These constraints encompass both theoretical restrictions and practical challenges that may be encountered, as detailed below:

Theoretically, LSTM model relies solely on historical price data for training and testing, and does not account for a deeper understanding of the factors behind price fluctuations, such as changes in the supply chain (for example, increased transportation costs, the uncertainty of agricultural production, etc., that may lead to price changes). Moreover, the model may not accurately predict price fluctuations caused by unforeseen events, such as natural disasters, political incidents, economic crises, shifts in consumer behavior and preferences, a sudden surge in the popularity of a certain vegetable due to health trends, or aversions caused by food safety issues, all of which could lead to rapid shifts in demand and pricing. If only historical price data is utilized, these shifts in consumer behavior cannot be captured.

In practical, vegetable prices are affected by a variety of complex factors, including seasonality, climate change, market supply and demand, shifts in government policy (such as adjustments to agricultural subsidies, changes in import and export tariffs), market intervention measures (such as minimum price guarantees or price control measures) and so on. Additionally, these factors may have differing impacts across different years. If data are not updated in a timely manner, the model may fail to capture the latest market trends, potentially leading to lagged predictions. Furthermore, due to data constraints, the current research only involves vegetable price data from wholesale markets in Beijing and does not cover national data or comparison with vegetable price predictions in other regions.

Considering potential future work directions, from a regional perspective, future studies could explore vegetable price predictions in different areas; from a temporal scale, they could compare whether there are differences in the short-term versus long-term predictions of vegetable prices in the same region and attempt to understand the underlying dynamic mechanisms.

Given that vegetable prices are influenced by a myriad of factors, future efforts could aim to further refine and optimize the model. Incorporating additional factors that affect price volatility, such as weather conditions, holiday effects, and supply chain changes, combined with historical price data and other explanatory variables, could utilize multi-model or mixed-model approaches to enhance predictive capabilities. It is hoped that through ongoing research and model improvement, more accurate forecasting tools can be provided for market participants and policymakers.

## Supporting information

S1 File (XLSX)
